# Engineering Bifunctional Catalytic Microenvironments for Durable and High-Energy-Density Metal–Air Batteries

**DOI:** 10.1007/s40820-025-01799-w

**Published:** 2025-06-13

**Authors:** Jean Marie Vianney Nsanzimana, Lebin Cai, Zhongqing Jiang, Bao Yu Xia, Thandavarayan Maiyalagan

**Affiliations:** 1 Department of Industrial Engineering, University of Padova, Via Marzolo 9, 35131 Padua, PD Italy; 2 Hubei Key Laboratory of Material Chemistry and Service Failure, Key Laboratory of Material Chemistry for Energy Conversion and Storage, Ministry of Education, School of Chemistry and Chemical Engineering, Huazhong University of Science and Technology (HUST), 1037 Luoyu Rd, Wuhan, 430074 People’s Republic of China; 3 Zhejiang Key Laboratory of Quantum State Control and Optical Field Manipulation, Department of Physics, Zhejiang Sci-Tech University, Hangzhou, 310018 Zhejiang People’s Republic of China; 4 Electrochemical Energy Laboratory, Department of Chemistry, SRM Institute of Science and Technology, Kattankulathur, Tamilnadu 603203 India; 5 Center for Next-Generation Energy Materials and School of Chemical Engineering, Sungkyunkwan University (SKKU), 2066, Seobu-ro, Jangan-gu, Suwon, Gyeonggi-do, 16419 Suwon, Korea

**Keywords:** Electrocatalysis, Earth-abundant materials, Bifunctional electrocatalysts, Oxygen electrocatalysis, Metal–air batteries

## Abstract

Rechargeable metal–air batteries have gained significant interest due to their high energy density and environmental benignity. However, these batteries face significant challenges, particularly related to the air-breathing electrode, resulting in poor cycle life, low efficiency, and catalyst degradation. Developing a robust bifunctional electrocatalyst remains difficult, as oxygen electrocatalysis involves sluggish kinetics and follows different reaction pathways, often requiring distinct active sites. Consequently, the poorly understood mechanisms and irreversible surface reconstruction in the catalyst’s microenvironment, such as atomic modulation, nano-/microscale, and surface interfaces, lead to accelerated degradation during charge and discharge cycles. Overcoming these barriers requires advancements in the development and understanding of bifunctional electrocatalysts. In this review, the critical components of metal–air batteries, the associated challenges, and the current engineering approaches to address these issues are discussed. Additionally, the mechanisms of oxygen electrocatalysis on the air electrodes are examined, along with insights into how chemical characteristics of materials influence these mechanisms. Furthermore, recent advances in bifunctional electrocatalysts are highlighted, with an emphasis on the synthesis strategies, microenvironmental modulations, and stabilized systems demonstrating efficient performance, particularly zinc– and lithium–air batteries. Finally, perspectives and future research directions are provided for designing efficient and durable bifunctional electrocatalysts for metal–air batteries.

## Highlights

- Overview of metal–air batteries architecture, reaction mechanisms, and challenges in developing bifunctional air-breathing electrodes.
- Comprehensive discussion on engineering the microenvironment chemistry of noble metal-free bifunctional oxygen electrocatalysts.
- Insights into future research directions for earth-abundant bifunctional catalysts with enhanced performance and durability, aiming to guide the future development of advanced bifunctional catalysts for scalable applications.

## Graphical abstract

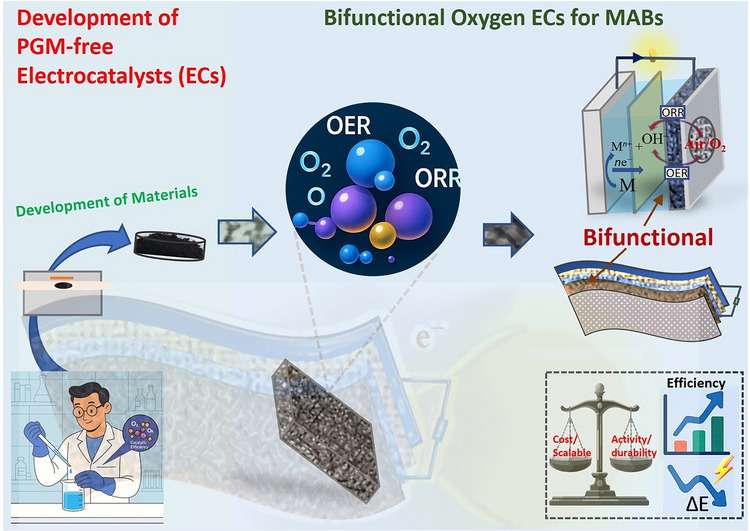

## Introduction

The increasing global energy demand and the urgency for transition to a decarbonized economy have paved new trends in electrochemical energy storage and conversion devices, including supercapacitors, fuel cells, water electrolyzers, and batteries, to play a key role toward a green, clean, and sustainable energy economy. These clean electrochemical energy technologies can make a bridge between the demand and supply of electricity produced from intermittent renewable energy sources and thus be used in many economic sectors like communication and transportation [1]. The primary pillar for electrochemical energy storage is the battery, which allows the storage of chemical energy and can be used, when necessary, in stationary stations or portable devices, making the battery a very pivotal device in daily needs. Although there has been significant progress in battery development, such as Li–ion batteries (LIBs) powering the electric vehicles (EVs), there is a big challenge to address due to the increasing demand for these devices. The raw materials resources’ long-term availability and geographical distribution of crucial metal elements for LIBs, which rely on scarce and finite resources such as cobalt and lithium, are limiting factors for future utilization of LIBs [2]. Additionally, safety concerns such as uncontrollable flammability in the case of using LIBs at a mass scale, recycling or disposal of materials after LIBs end-of-life pose a significant challenge to satisfy the needs of environmental-friendly technologies and ever-increasing energy storage [3]. To get a socioeconomic credence for the battery technology, we need to showcase high power and energy density coupled with cost-effectiveness. For this, rechargeable metal–air batteries (MABs) are used in a wide range of applications due to their low cost compared to LIBs. The MABs also offer favorable properties and practicability, including relatively high energy density and non-flammable electrolytes, which ensure high safety [4–7].

The main aspect of MABs that makes them an appealing energy storage alternative to LIBs is its reduced weight due to the utilization of atmospheric oxygen as the reactant. This fuel of MABs is environmentally benign, making MABs a clean technology [8–10]. Furthermore, there is a possibility of using several metal elements at the anode, such as first group metals including lithium, sodium, and potassium, in second group metal elements like magnesium and calcium can be used as well as from the third group metal like aluminum and a few transition metals like iron and zinc. Most of these metals, which can be adapted in the MABs, are of relatively low cost, earth-crust abundant, eco-friendly, non-toxic, and a viable alternative [11]. Due to the use of air as a fuel, the electrocatalysts play a pivotal role in oxygen electrocatalysis and thus contribute to the performance, making them at the corner of developing high-performing and durable MABs [12]. However, catalysts in the operating conditions undergo reversible electrode reactions. The continued redox cycling affects the microenvironment, accelerating catalyst degradation, including active site dissolution, irreversible surface reconstruction, and subsequent crystal structure collapse, which ultimately leads to a sharp decay in battery cycling performance. It makes a great challenge to develop redox-tolerant materials capable of operating in this diverse microenvironment chemistry.

Despite the high performance of precious metal catalysts for oxygen reduction reaction (ORR) and oxygen evolution reaction (OER), their bifunctional activities are also hindered by the intrinsic inertness in oxidation state modulation. Transition metals possess a wider space of tunable d-orbital electronic structures, enabling adaptive valence transition during redox processes, resulting in bifunctional adsorption energies for multiple oxygen-containing intermediates. Therefore, the search for valence-switchable transition metals should be a priority in the research and development of materials for cathodic applications in long-term, safe MABs. Numerous progresses have witnessed the potential of bifunctional transition-metal catalysts for MABs; however, most focus on the activity optimization via doping metastable metals as electron buffers, introducing multiple synergistic sites, constructing heterostructures, etc. The structure-dependent stability during redox reactions is still uncontrollable. The stabilized mechanism of different regulatory strategies on structure still lacks systematic research and identification. This review describes the operating components and their challenges in terms of basic MAB principles. The OER/ORR reaction mechanisms of key air-breathing electrodes are further clarified. In addition, this review emphasizes summarizing the recent progress on various bifunctional PGMs-free metal catalysts, focusing on the discussion of microenvironment structure-dependent stability and the intrinsic regulation mechanisms, which provides insight into the future development of redox-tolerable catalysts for high-performance MABs.

## Metal–Air Battery Configurations, Operating Principles, and Challenges

**Fig. 1:**
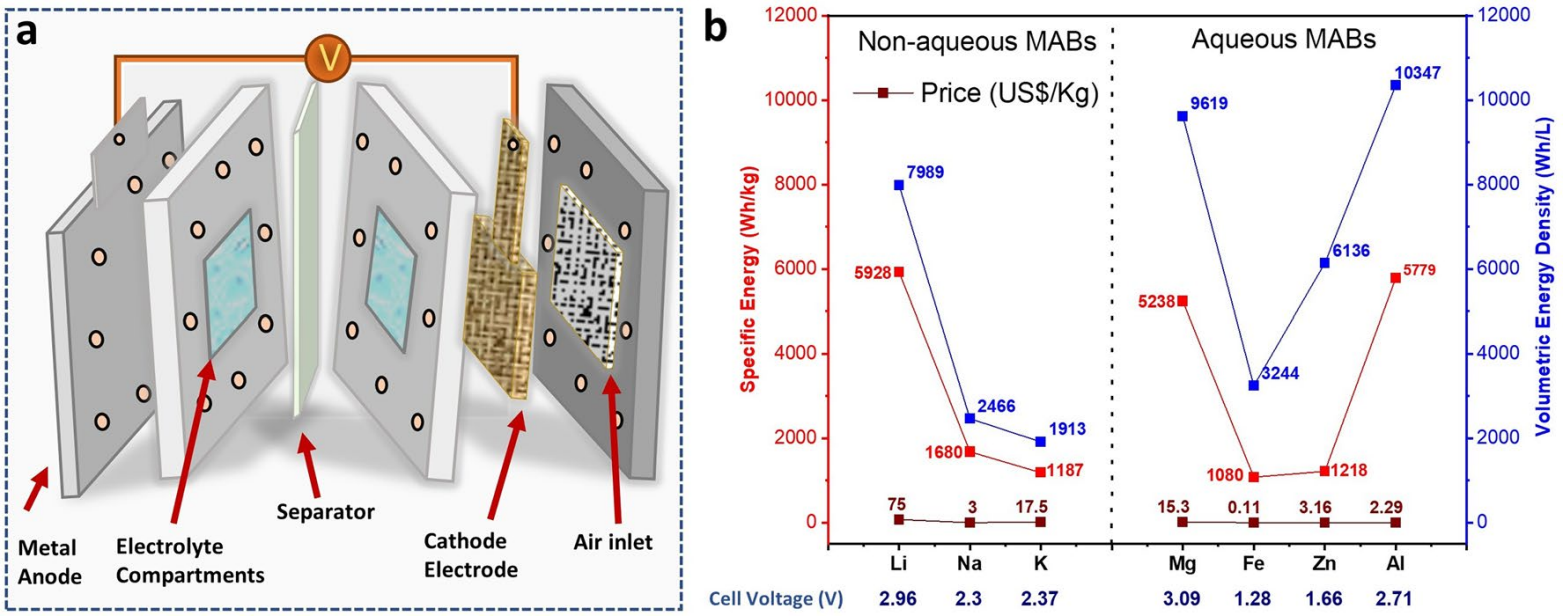
**a** Schematic representation of MABs. **b** A diagram comparing Li, Na, K, Mg, Fe, Zn, and Al metal anodes in MABs. The OCV values are reported along the metal elements on the x-axis. This comparative diagram also depicts the specific energy density, volumetric energy density, and price in US dollars per kilogram

As schematically illustrated in Fig. 1a, MABs consist of several key components, each serving a specific function and contributing to the device’s performance and durability. Typically, a MAB cell consists of four main components: a metal anode, an air-breathing cathode, a membrane separator, and an electrolyte [13, 14]. In addition to these major components, other components such as current collector and fuel access ports to allow the air in the device, work together to enable the battery to generate energy via the chemical oxidation of the metal at the anode and the reduction of oxygen at the porous cathode, during the discharge, while the electrolyte allows for the flow of ions between them. This section mainly introduces critical roles of various components and their optimizing strategies to address current challenges.

### Metal Anode

The metal anode is the metal that undergoes oxidation during discharge, and the name of a MAB is derived from the metal anode [7]. During the discharge, the metal anode releases electrons due to the oxidation reaction and flows through the external circuit, providing electrical energy. Due to the growing interest in MABs, several types of metals that can be used as metal anodes, such as zinc, lithium, aluminum, magnesium, iron, sodium, and potassium, have been explored [13, 15]. Nevertheless, the energy density of MAB is likely to be related to the metal used as an anode, and this makes metal anode a critical component in MABs. Considering the pure metal anodes, the magnesium–air battery in which the metal anode is magnesium demonstrated a higher open-circuit voltage (OCV) (Fig. 1b), while the iron–air battery delivers a reduced OCV of 1.28 V (298 K), and it is the cheapest metal elements among the mostly used metal elements as anode [16, 17]. Although the magnesium–air batteries exhibit the highest OCV of 3.09 V and high specific energy density, their high corrosion characteristics, unlike other anode metals, where the increase in cathodic current unexpectedly accelerates hydrogen evolution, remain a major challenge [18]. Additionally, their poor reversibility due to the magnesium oxide (MgO) and magnesium peroxide (MgO_2_) as by-products during discharge passivating the electrode demonstrates sluggish charge transport and poor conductivity. These impede the battery’s rechargeability and performance, which is a crucial parameter for the practical commercialization of MABs [19]. Despite iron being the cheapest and one of the most abundant metals in Earth’s crust among these metal anodes for MABs, it displays the lowest theoretical specific energy [17]. Among the other challenges, the poor stability of the iron anode in aqueous iron–air batteries is critical due to surface passivation by iron oxide species and volume changes during cycling, causing mechanical strain [20]. Due to the low expected capacity and the expected working voltage, it shows a low prospect for commercialization compared to zinc– and lithium–air batteries (LABs).

For practical application, zinc–air batteries (ZABs), with OCV of 1.66 V [14], and a specific energy of 1218 Wh kg^−1^, and lithium–air batteries (LABs), with a specific energy of 5928 Wh kg^−1^ and a higher OCV of 2.96 V, have received great attention [21, 22]. The properties of MABs, such as OCV, depend primarily on the intrinsic properties of metal anodes [22]. Additionally, the anode is prone to dendritic growth, where sharp metallic protrusions can pierce the separator, leading to internal short circuits. Parasitic chemical and electrochemical corrosion reactions also occur even when the battery is idle, causing self-discharge and reducing efficiency [23]. These issues necessitate optimizing strategies, such as the architecture of the electrode, composition optimization of the anode by alloying strategy, coating, and electrolyte engineering, for enhanced intrinsic activity and durability [23, 24].

As the dendrites’ growth is a critical issue, to suppress dendrite growth on the Li anode, Ma B. et al., developed a hydrophobic Li^+^-solvated structure [25]. The functional group of hexafluoroisopropyl acrylate, used as an additive, enabled the formation of a stable solid–electrolyte interface (SEI). As a result, dendrite growth was suppressed, and the overall battery performance was improved. Furthermore, the introduction of functional inorganic additives in composite solid-state electrolytes has proven to be an effective approach to enhancing multiple aspects of flexible LIBs, such as boosting ionic conductivity, suppressing dendrite formation, and improving both safety and stability [26]. Furthermore, the alloying strategy, such as in aluminum–air batteries, not only minimizes the corrosion of the anode but also improves overall performance [27]. This highlights the promise of alloying; however, the mechanism behind this corrosion minimization, as well as its effect on activity and long-term stability, requires further investigation.

### Electrolyte and Separator

Electrolytes in MABs play a critical role in facilitating the electrochemical reactions and the stability of both the metal anode and the air cathode. Depending on the type of MABs, the electrolyte could be non-aqueous aprotic electrolytes in Li–, Na–, and K–O_2_ batteries, or aqueous liquid electrolytes in Zn–, Al–, Mg–, and Fe–air batteries [28]. In non-aqueous batteries, non-aqueous aprotic organic ethers are used, but they are highly sensitive to external environmental conditions. Therefore, the assembly must be prepared in an inert environment, and they are typically tested using pure oxygen [29]. Non-aqueous electrolytes, such as organic solvents or ionic liquids, offer significant advantages in their broader electrochemical window, which allows operation at higher voltages (3–4 vs. ~ 1.23 V for water), simultaneously minimizing parasitic reactions, such as hydrogen evolution, that corrode metal anodes in aqueous electrolytes. However, they suffer from decomposition during cycling, leading to electrolyte depletion and resistive by-products. Aqueous MABs employ either aqueous electrolytes (e.g., alkaline aqueous electrolytes) or solid-state electrolytes (e.g., alkaline gel polymer electrolytes), in flexible aqueous MABs [29]. Although the aqueous electrolytes are cost-effective, have high ionic conductivity, and do not require an inert environment during fabrication and testing, they face evaporation and carbonate formation from airborne CO_2_. With the rising interest in flexible MABs for wearable electronics, their electrolytes should exhibit enhanced flexibility, robust mechanical and chemical stability. These are prepared by encapsulating aqueous electrolytes, such as highly concentrated alkaline solutions, polymeric matrices like polyacrylonitrile and polyacrylamide [30]. These hydrogel electrolytes face challenges related to ionic conductivity, cation mobility at the anode, and electrochemical stability. However, emerging approaches have shown promise in mitigating these issues through modified hydrogel electrolytes. For example, cationic hydrogels with long cationic branch chains within the polymeric matrix create new ion transport pathways. This enhancement improves reversibility, increases ionic conductivity, and enhances electrochemical stability [4].

Electrolytes play a critical role in addressing the challenge of dendrite formation and the side reaction of the anode electrode even before the operation [31]. For example, Jiang Zhou’s group demonstrated an approach to suppress dendrites on a zinc anode through the microlevelling effect [32]. This effect was induced by the inclusion of metallic cations (Gd^3+^ ions) in the conventional electrolyte. The presence of these cations in the electrolyte triggered their adsorption on the zinc anode and modulated the microlevelling effect, thereby enabling dendrite-free zinc anodes due to the enhanced reversibility (plating and stripping) of zinc during operation. This shows that additives in the electrolytes are an efficient method to boost the performance of MABs and suppress the formation of dendrites at the anode [32, 33]. In addition, a separator characterized as a microporous polymer membrane is placed in the electrolyte to physically isolate the anode and cathode while permitting ionic conduction [34]. Its integrity is vital to prevent short circuits, yet dendrite penetration and chemical degradation by the electrolyte pose risks. Enhancing the separator’s mechanical strength and chemical inertness is crucial, especially in rechargeable systems where repeated cycling exacerbates wear.

### Air Cathode

**Fig. 2:**
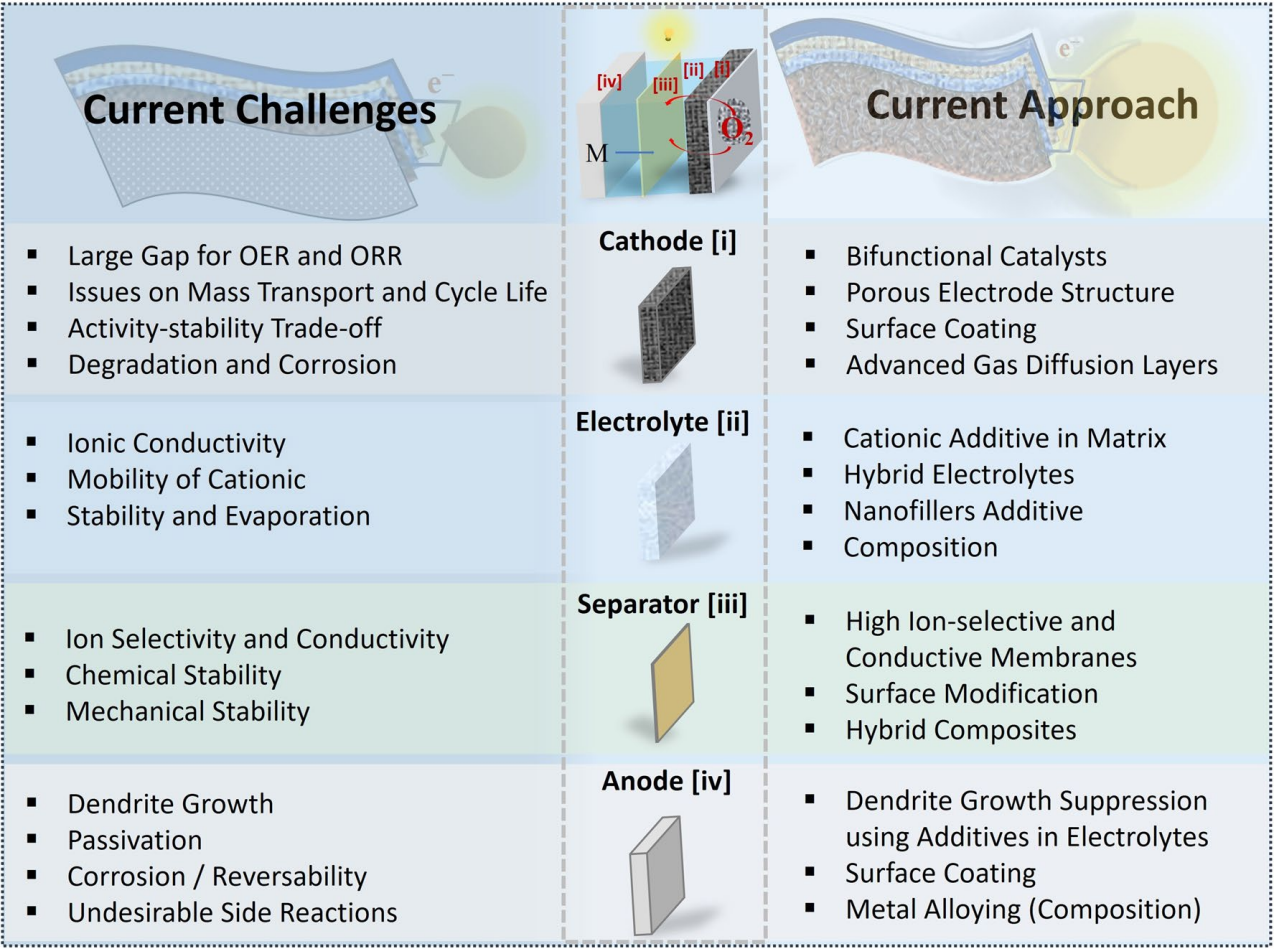
Scheme depicting the major parts of MABs, associated challenges, and current approaches to address these issues

Oxygen as a fuel for MABs is supplied on the positive electrode (air cathode, Fig. 1a), where a catalytically active layer performs two key electrochemical reactions: OER and ORR. To facilitate the diffusion of O_2_ from the air to the layer catalysts, the cathodes are usually fabricated as porous gas diffusion layer (GDL) or on porous materials like metal foams to deliver excellent conductivity, expose a large surface area, and enhance transport [16]. At this oxygen electrode, ORR takes place during the discharge, while OER occurs during the charging [13]. Both oxygen reactions are multistep reactions and exhibit sluggish kinetics, thus limiting the performance of MABs [11]. Bifunctional catalysts must drive both OER during charging and ORR during discharge; however, few materials durably meet the demanding requirement. Holistic material innovations are urgent for the switchable bifunctional activity and redox stability. Beyond catalysts, the continuous operation of the devices also faces significant challenges. For instance, the infiltration of environmental contaminants such as moisture and CO_2_, which can form carbonates in the electrolyte, impairs performance. Furthermore, during discharge, reaction products may accumulate in the cathode pores, causing clogging and limiting oxygen diffusion, thereby reducing capacity over time. All these issues should be addressed through systematic material design and engineering to synergistically enhance the performance of the MABs (Fig. 2).

## Fundamentals of Chemical Reactions and Challenges in MABs

**Fig. 3:**
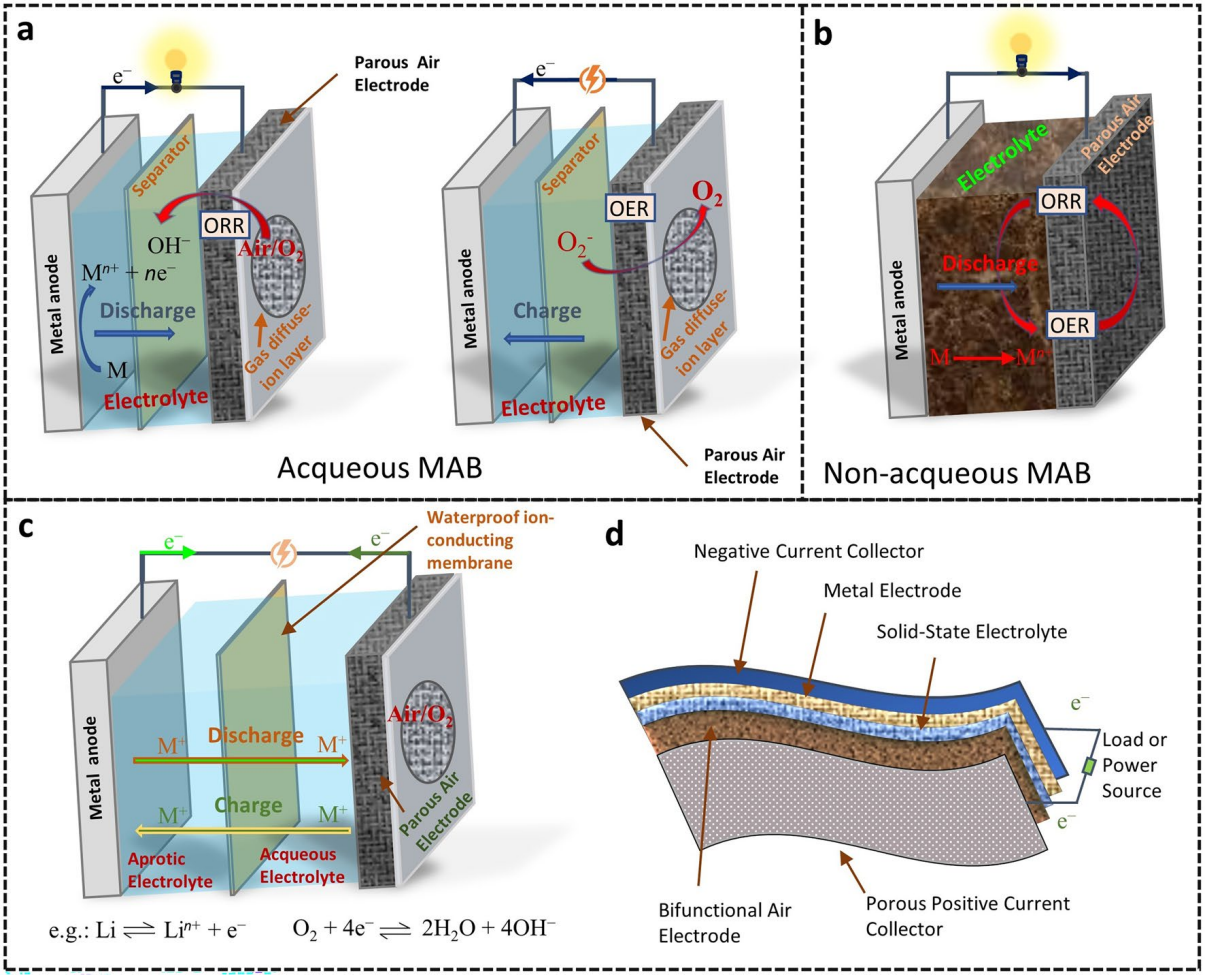
Schema depicting the operation principle of MABs. **a** Aqueous (Zn‐O_2_), **b** non-aqueous (e.g.: Li‐O_2_, K‐O_2_, and Na‐O_2_ batteries), **c** solid‐state/aqueous hybrid, and **d** flexible MABs

In MABs, the main types of chemical reactions can be categorized based on different electrolyte systems as well as the metal anode [7]. The electrolyte is an essential critical component enabling the design of various types of MABs (aqueous, non-aqueous, and solid-state), including those requiring mechanical stability, such as flexible MABs (Fig. 3). For example, Eqs. 1 and 2 depict the typical chemical reactions in Li–, K–, and Na–air batteries with aprotic electrolytes [34]. At the cathode, the O_2_ from the air reacts with metal ions, resulting in the discharge of metal superoxides or peroxides on the air electrode (Eq. 2). In aqueous electrolytes, the O_2_ from the air at the cathode undergoes a chemical reduction reaction by accepting electrons, forming OH^−^. Afterward, the formed OH^−^ interacts with metal ions from the anode (Eqs. 3 and 4) [15, 34]. Nevertheless, tremendous efforts have been devoted to developing highly performing MABs in aqueous electrolytes due to the high operation safety, great ionic conductivity, and low cost [35].

**Non-aqueous electrolyte:**

1 $${\text{Metal electrode:}}\,{\text{M}} \leftrightarrow {\text{M}}^{ + } + {\text{ e}}^{ - }$$

2 $${\text{Air electrode:}}\,{\text{xM}}^{ + } + {\text{O}}_{{2}} + {\text{ xe}}^{ - } + {\text{ e}}^{ - } \leftrightarrow {\text{M}}_{{\text{x}}} {\text{O}}_{{2}}\, \left( {{\text{x }} = {\text{ 1 or 2}}} \right)$$

**Aqueous electrolyte:**

3 $${\text{Metal electrode:}}\,{\text{M}} \leftrightarrow {\text{M}}^{{{\text{n}} + }} + {\text{ ne}}^{ - }$$

4 $${\text{Air electrode:}}\,{\text{O}}_{{2}} + {\text{ 4e}}^{ - } \leftrightarrow {\text{ 2H}}_{{2}} {\text{O}}_{{2}} + {\text{ 4HO}}^{ - }$$

where M represents the metal (Al, Zn, Mg, Fe, etc.) and n stands for the charge number of metal ions.

Although there has been promising advancement in MABs, numerous challenges remain. The lack of materials with accelerated kinetics for the two key chemical reactions (ORR and OER) is a significant bottleneck that limits the charge and discharge efficiency [36]. It is crucial to identify the reverse reaction pathways during switchable OER and ORR, which limit the bifunctionality of catalysts without causing severe degradation. However, since the chemical mechanisms of these two reactions differ, this effort is particularly challenging. This section systematically summarizes these intrinsic mechanisms for both OER and ORR, shedding insight into the design of advanced bifunctional catalysts for switchable ORR and OER during the cycles in MABs.

The reduction of O_2_ at the air electrode is still a major issue in MABs. This reaction occurs at the triple-phase interface, where the catalyst (solid state) layer simultaneously interacts with the gaseous O_2_ and electrolyte [37]. Due to the sluggish kinetics of this reaction, the air-breathing electrode has become the performance-limiting electrode in MABs. The best material for ORR is the PGM-based catalysts, such as platinum, and however, the scarcity of PGMs makes them unapplicable on a large scale to satisfy the ever-increasing demand of MABs. Thus, it is crucial to develop PGM-free catalysts that use a limited amount of these scarce metal elements. Additionally, ORR and OER occur at the same electrode, which makes it a challenge even for PGMs in the MABs, as they often lack bifunctional catalytic activity. Hence, more intensive effort is required to develop catalysts beyond the PGMs for air-breathing electrodes.

**Fig. 4:**
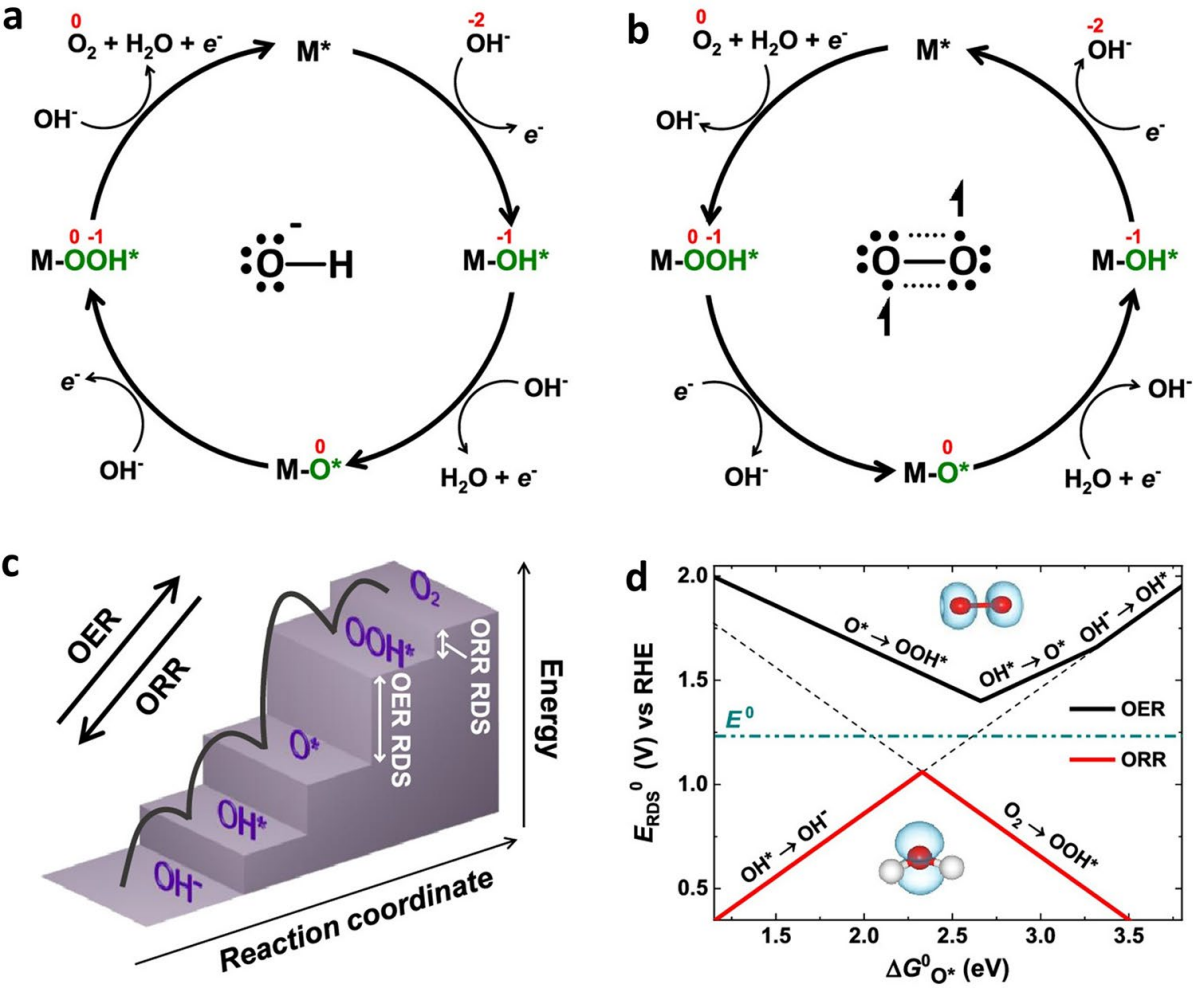
Oxygen electrocatalysis mechanisms and diagrams; **a**,** b** Elementary chemical reactions corresponding to the chemical steps during OER and ORR cycles in alkaline conditions are illustrated. **c** Diagram depicting the potential energy for a catalyst at a potential E = 0 V vs. RHE.** d** Schematic representation of activity volcano plot of the rate-determining steps for ORR and OER. Reproduced with permission [38]. Copyright 2019, American Chemical Society

The ORR may proceed through either two- or four-electron reaction pathways in aqueous electrolytes. The four-electron pathway (Eqs. 5–8) with E_0_ =  + 0.401 V produces water (Fig. 4a) [38], whereas the two-electron mechanism with E_0_ =  − 0.076 V results in the formation of hydrogen peroxide (Eqs. 10 and 11) [11]. The hydrogen peroxide is not a desirable pathway as it may lead to corrosion of some materials in the MABs or fuel cells [39].

5 $${\text{M}}^{*} \, + {\text{ O}}_{{2}} + {\text{ H}}_{{2}} {\text{O }} + {\text{ e}}^{ - } \to {\text{ OOHM}}^{*} \, + {\text{ OH}}^{ - }$$

6 $${\text{OOHM}}^{*} \, + {\text{ e}}^{ - } \to {\text{ OM}}^{*} \, + {\text{ OH}}^{ - }$$

7 $${\text{OM}}^{*} \, + {\text{ H}}_{{2}} {\text{O }} + {\text{ e}}^{ - } \to {\text{ OHM}}^{*} \, + {\text{ OH}}^{ - }$$

8 $${\text{OHM}}^{*} \, + {\text{ e}}^{ - } \to {\text{ OH}}^{ - } + {\text{ M}}^{*}$$

The symbol ∗ denotes the catalyst’s active site, while OOH^ *^, O^ *^, and OH ^ *^ represent intermediates adsorbed onto these active sites, which may appear as deprotonated species such as O_2_^–^ and O^–^, respectively . Undesirable molecular oxygen, which is the fuel of MABs, can be electrochemically reduced in a two 2e^−^ routes, forming a solo intermediate of OOH^*^ and leading to the formation of H_2_O_2_ as the product species (see Eqs. 9, 10 and Fig. 4b) [40]:

9 $$^{*} \, + {\text{ O}}_{{2}}^{ } + {\text{H}}\hbox{-}_{{2}} {\text{O }} + {\text{e}}^{ - } \to {\text{OOH}}^{*} + {\text{ OH}}^{ - }$$

10 $${\text{OOH}}^{*} + {\text{ e}}^{ - } \to ^{*} + {\text{ OH}}_{{2}}^{ - }$$

The OER exhibits slow kinetics and involves multiple electron transfer processes, resulting in the formation of various intermediates [41]. The mechanism of OER under alkaline conditions can be summarized in Eqs. 11–14 [42]. The well-known OER mechanism is the adsorbate evolution mechanism (AEM), in which oxygen-containing adsorbates participate in catalytic redox reactions on the metal active sites [43]. There are two possible pathways for OER: the first proceeds through Eqs. 11–13, while the other involves the formation of M^*^OOH^−^ as an intermediate and reacts with OH^−^ ions to generate O_2_ molecule (Eqs. 14 and 15) [42, 44]. The oxygen evolution activity at the metal active sites depends on the interaction with oxygen intermediate species (see Eqs. 11, 12, 14), during the multistep process [43]:

11 $${\text{M}}^{*+} + {\text{ OH}}^{ - } \to {\text{ M}}^{*}{\text{OH }} + {\text{ e}}^{ - }$$

12 $${\text{M}}^{*}{\text{OH }} + {\text{ OH}}^{ - } \to {\text{ MO }} + {\text{ H}}_{{2}} {\text{O }} + {\text{ e}}^{ - }$$

13 $${\text{2MO }} \to {\text{ 2M }} + {\text{ O}}_{{2}}$$

14 $${\text{MO }} + {\text{ OH}}^{ - } \to {\text{M}}^{*}{\text{OOH }} + {\text{ e}}^{ - }$$

15 $${\text{M}}^{*}{\text{OOH }} + {\text{ OH}}^{ - } \to {\text{ M }} + {\text{ O}}_{{2}} + {\text{ H}}_{{2}} {\text{O }} + {\text{ e}}^{ - }$$

Although the mechanism involves four-electron process which might look like a reversible reaction between ORR and OER (Fig. 4c), the Volcano plot of scaling relationship in Fig. 4d reveals that these two reactions show mismatch in oxygen adsorption energy and thus making it difficult for catalysts to perform well in both two reactions [38]. Thus, breaking the linear-scale relationship by rational structural design is the key to realizing high-activity bifunctional catalysts that can bridge this mismatch of most optimized intermediate adsorption in different reactions.

The catalyst structural features influence the activity and reaction pathways of OER and ORR. For instance, using cobalt with different geometric sizes and coordination environments was reported to efficiently tune the ORR in both 2- and 4-electron pathways [45]. The Co nanoclusters in cobalt SAC and nanoclusters on nitrogen-doped hierarchical porous carbon favored the four-electron pathways, while the Co SAC selectively favors two-electron mechanism. This difference further underscores the critical influence of active site microenvironment on the oxygen electrolysis. By adjusting material structural features such as the electronic structure, defect density, coordination environment, or crystal facet, one can switch or favor a particular pathway (AEM vs LOM), leading to distinct reaction energetics and kinetics. For example, the doping of a small amount of Mo (∼0.5 wt%) modifies the AEM mechanism of Co_3_O_4_ during OER to a fast oxide path mechanism (OPM) due to the creation of oxygen vacancies by doping Mo, which activates the lattice oxygen after leaching from the lattice of the structure [46]. While the AEM is limited due to its required theoretical overpotential (0.37 V), recently, there has been interest in tuning alternative OER mechanisms through microstructural modification [47]. For instance, modifying the B-site metals in ABO_3_, such as LaNiO₃ perovskites, enhances metal–oxygen covalency, leading to the formation of lattice oxygen vacancies and consequently shifting the OER mechanism from the AEM to the LOM pathway [48]. Although the AEM mechanism is dominant in perovskites, it was demonstrated by theoretical studies that the OER mechanism and activity have a relationship with the structure of ABO_3_ perovskites, where the modulation of A cation sites enables the LOM [49]. As the doping of cations at either A cation or B cation sites or even O-site induces structure changes and as a consequence transform the oxygen electrolysis mechanisms and activity, engineering approaches of modifying the microenvironmental structure such as electronic structure, crystallinity, redox chemistry, vacancies, doping, strains, and interfaces would influence the OER and ORR activity [47].

## Synthesis Methods of Electrocatalysts for MABs

Recent advancements in the synthesis of functional materials have opened new pathways for developing diverse nanostructured systems with finely tuned microenvironments at the atomic, nano/micro scale, and interfaces, thereby enhancing their catalytic properties, including bifunctional activity for OER and ORR. For instance, in efforts to minimize metal usage, innovations in synthetic strategies have enabled the fabrication of single-atom catalysts (SACs) with high dispersion on carbon or metal supports, ensuring optimal utilization of active sites. They are various strategies for synthesizing SACs and dual-atom catalysts (DACs), including (1) dry synthesis route (atomic layer deposition (ALD) method, pyrolysis synthesis, atom trapping method, and two-step doping method); and (2) wet synthesis route taking advantageous on adsorption of the atoms on the substrate (facile adsorption method, wetness impregnation method, and strong electrostatic adsorption method) or photochemical and electrochemical properties of SAC with substrate (e.g.: photoreduction method) [50]. Metal–organic frameworks (MOFs), due to their well-defined structure and tunable porosity, serve as ideal precursors for the synthesis of relatively scalable SACs. Upon pyrolysis, organic precursors including MOFs, metal–phenanthroline complexes, and metal phthalocyanine–silica colloid composites decompose thermochemically, resulting in metal–nitrogen–carbon SACs and DACs [51]. Additionally, MOF pyrolysis can be employed to synthesize metal alloys supported on nitrogen-doped carbon [52]. Although these techniques are promising for SAC catalysts synthesis, the economical and high-efficiency synthesis hampers large-scale industrialization [53]. Hence, there is a need to investigate the cost-effectiveness of these methods for actual MABs and make an effort to use a reproducible and scalable approach.

Furthermore, hydrothermal synthesis, whether via a one-pot process or coupled with subsequent heat treatment, has emerged as a promising route for producing metal alloys, metal hydroxides, metal oxides, and metal chalcogenides. This method enables control over microenvironment chemistry and structural phases through modulation of nucleation and growth kinetics. Some of the hydrothermal synthesis is practical for large-scale production as the conditions can be controlled, while enabling the reproducibility and scalability of the products. Furthermore, self-assembly, chemical, and electrochemical approaches, such as reproducible chemical reduction and electrodeposition, facilitate the formation of supported (self-supported) or support-free materials tailored for catalytic applications [54, 55]. For example, a chronopotentiostatic superoxidation was employed to fabricate a sulf-supported Ni electrode, and due to its three-electron transfers (Ni ↔ Ni^3+^), the constructed aqueous Ni–Zn batteries demonstrated an excellent energy density of 6.88 mWh cm^−2^ and flexibility with capable of powering a microcell [56]. Although these techniques have been established as efficient approaches, innovative and novel strategies are paramount for developing engineered microenvironmental chemistry to create durable and efficient bifunctional oxygen electrocatalysts. For example, Guowei Yang et al. developed a single-step bipolar doping strategy to synthesize Janus DACs based on Ni and Fe centers, which enhanced charge separation and showed superior performance in light-assisted rechargeable zinc–air batteries [57]. The tailored atomic microenvironmental chemistry of the dual Ni and Fe centers demonstrated efficient hole and electron enrichment sites, photoelectrochemical characteristics, and superior performance for both the OER and ORR. Additionally, the synthesis of SACs with low metal loading but large surface area is crucial for overall performance. For instance, boric acid (H_3_BO_3_) assisted in the one-pot pyrolysis of cobalt salt and chitosan, resulting in a hierarchical porous structure with a high specific surface area that exposed active sites for oxygen electrocatalysis, thereby achieving outstanding catalytic activity [58]. Due to the simplicity of using one-pot synthesis and the usage of abundant chitosan, this synthesis proved scalable and reproducible. As the synthesis strategy plays a crucial role in engineering the microenvironment and tuning the physicochemical and electrochemical properties, it is crucial to extend the search of novel synthesis approach and also combining with the advanced theoretical calculations such as first-principle calculations enabling the development of materials with desirable properties in electrochemical energy storage and conversion technologies and beyond [59].

## Bifunctional Oxygen Electrocatalysts

Although the operating principles for MABs are similar, the bifunctional electrocatalysts should be tailored to electrolyze oxygen, but should also be stable in the reversible reaction of oxygen oxidation and reduction. Thus, searching redox-tolerated materials should tailor the capability of long-term charge/discharge switching [37]. Although PGMs are the best ORR and OER electrocatalysts, they lack bifunctional catalytic activity for oxygen electrocatalysis. Transition metals possess a wider space of tunable d-orbital electronic structures, enabling adaptive valence transition during redox processes, resulting in bifunctional adsorption energies for multiple oxygen-containing intermediates. In this review, we will summarize recently developed PGM-free bifunctional catalysts for the air-breathing electrode from seven aspects: (i) single-atom catalysts, (ii) spinels, (iii) perovskites, (iv) metal-free catalyst, (v) metal–nitrogen–carbon, (vi) metal–organic frameworks-derived catalysts, (vii) alloys and high-entropy alloys.

### Single-Atom Catalysts

**Fig. 5:**
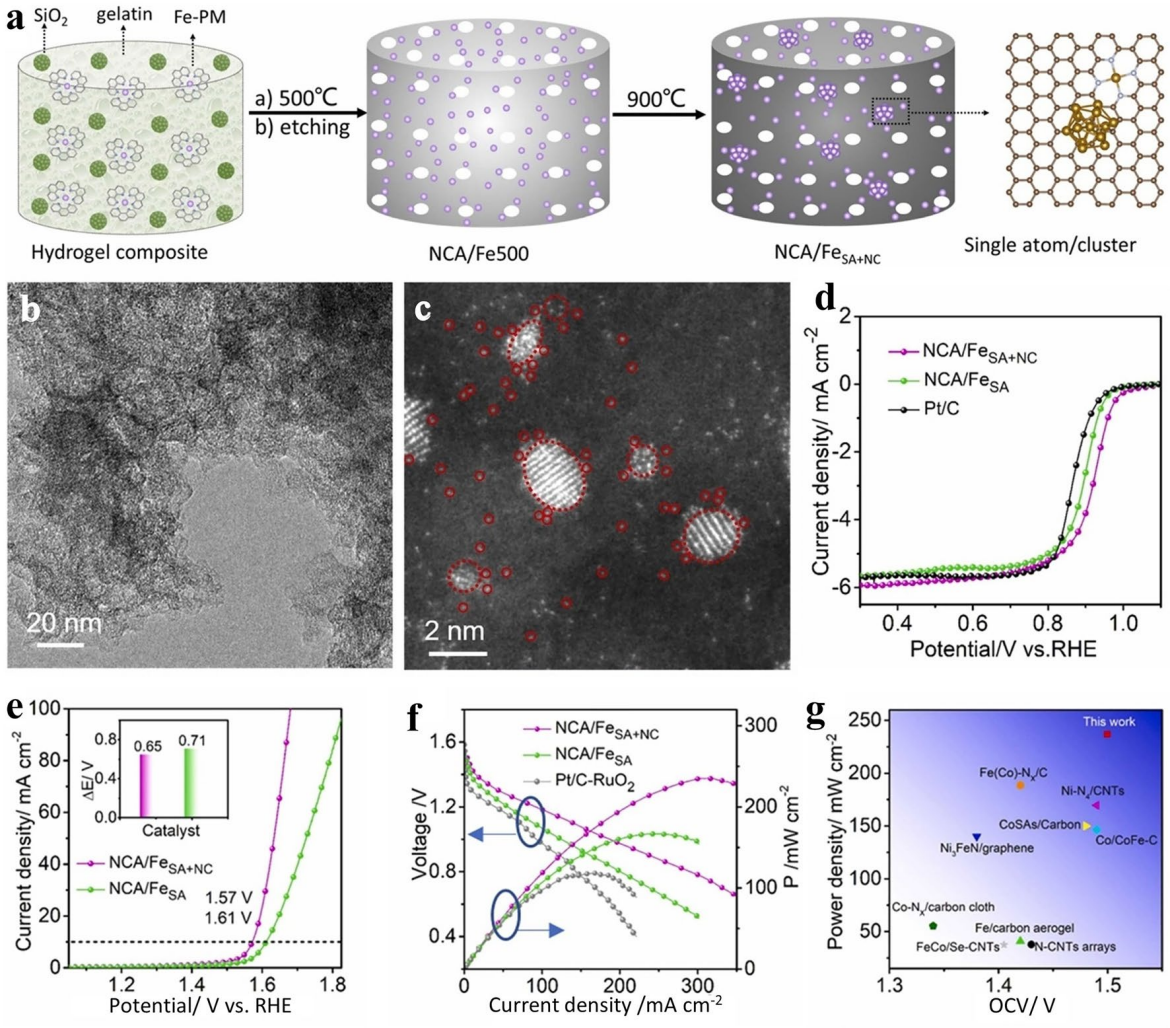
**a** Scheme summarizing the synthesis procedure of NCA/Fe_SA+NC_ electrocatalyst. **b**, **c** TEM image and corresponding HRTEM image showing the Fe SACs and Fe clusters. **d** ORR performance in 0.1 M KOH. **e** Polarization curves for OER in 1.0 M KOH. The inset in Fig. 5e depicts the ΔE of these two electrocatalysts. **f** Curves of constant-current discharging in zinc quasi-solid batteries. **g** Diagram comparing the optimized NCA/Fe_SA+NC_ electrocatalyst with selected ZABs. Reproduced with permission [63]. Copyright 2023, Elsevier

Recently, single-atom catalysts (SACs) have received great interest not only as an engineerable approach to reduce precious metal elements in electrocatalysis but also to develop highly performing materials, especially in electrochemical energy conversion and storage systems. As many electrochemical reactions in heterogeenous catalysis occur at the atoms as the catalytic active sites, the SACs offer a unique approach to push boundaries in catalysis, like to tune active sites and optimizing the selectivity [60]. Since the interest in SACs, there have been significant breakthroughs in terms of synthesis and potential application [61, 62]. To address the durability of SACs, coupling with nanoclusters was found to be a promising approach. For example, the Fe SACs with adjacent Fe nanoclusters were synthesized through a straightforward pyrolysis of biomass hydrogels, which served as Fe precursor and nitrogen-doped carbon (Fig. 5a) [63]. The study showed that the presence of Fe nanoclusters increased the 3d electron density and reduced the magnetic moment of Fe atomic centers (Fig. 5b, c), which improved the bifunctional catalytic properties and oxidation resistance of the FeN₄ sites compared to samples without nanoclusters (Fig. 5d, e). In a flexible ZAB, the NCA/Fe_SA+NC_ functioned as a bifunctional cathode catalyst at − 40 °C, delivering impressive performance with an OCV of 1.47 V, a power density of 49 mW cm⁻2 (Fig. 5f, g), and outstanding long-term performance, sustaining 2,300 continuous recharge/discharge cycles. Additionally, at ambient conditions, the NCA/Fe_SA+NC_ demonstrated enhanced catalytic activity, emphasizing the importance of tuning the electronic structure of SACs to tailor both performance and longevity.

**Fig. 6:**
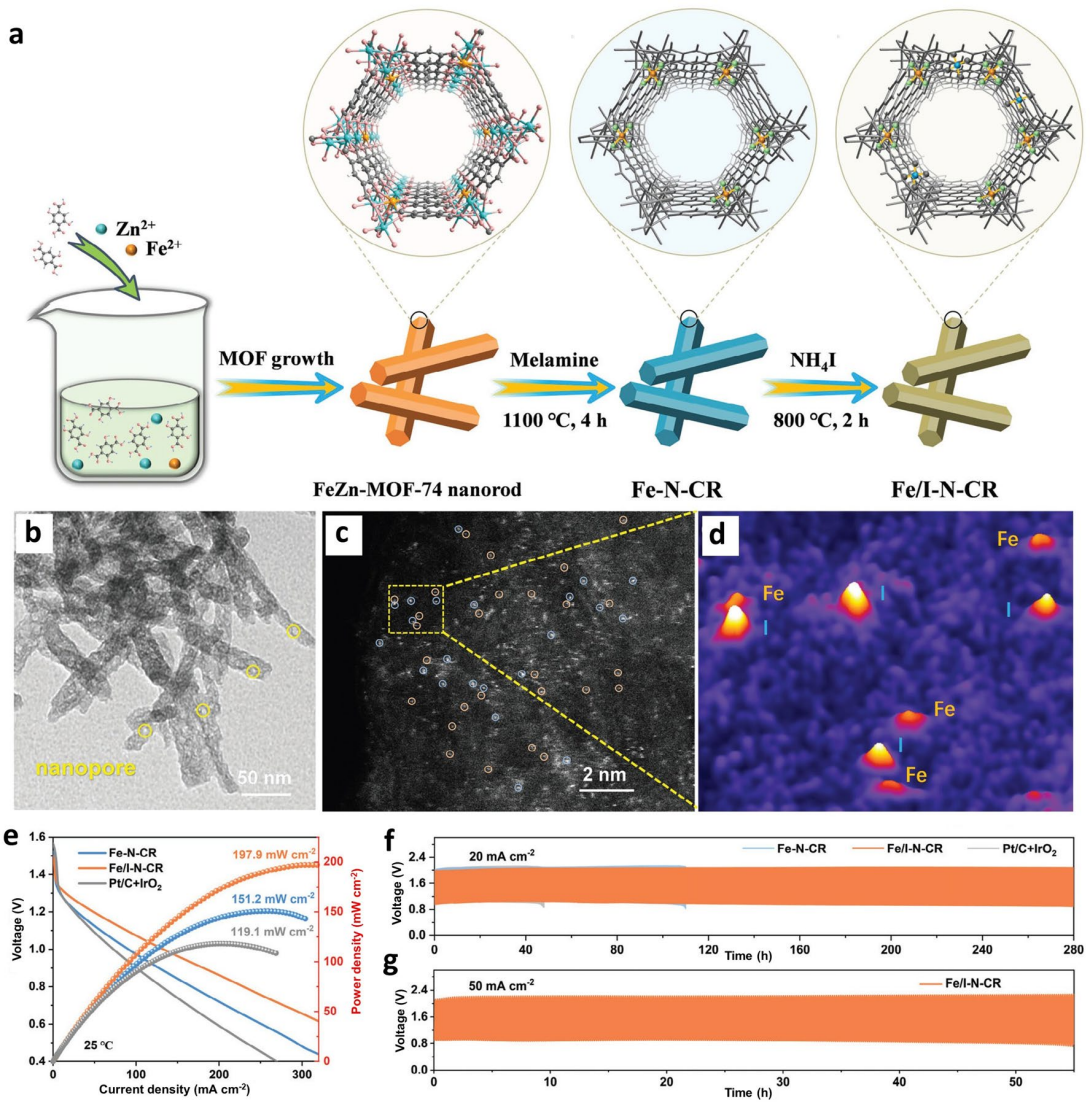
Microscopic analysis of Fe/I-N-CR: **a** Schematic illustration of Fe/I-N-CR fabrication process, **b**, **c** TEM images, **d** corresponding aberration-corrected HAADF-STEM images of Fe/I-N-CR with orange circles representing Fe atoms, and the blue circles represent I atoms. Performance evaluation: **e** Curves of discharge polarization and power density of assembled quasi-solid-state ZABs, **f**, **g** corresponding charging/discharging performance at 20 mA cm^−2^ and 50 mA cm^−2^. Reproduced with permission [51]. Copyright 2024, John Wiley and Sons

In addition to enhancing both the performance and stability of iron-based SACs by the electron spin-state transfer due to the presence of Fe atomic clusters, which enable the filling of σ ∗ orbital, thereby promoting OH − desorption and accelerating the reaction kinetics of the rate-determining step [64]. The synthesis of dual single-atom catalysts (DACs) was recently reported as an excellent approach to tailoring the advanced catalytic performance of SACs. Either adding a non-metal element or another metal element is a successful method to develop DACs [65]. For instance, Fe and iodine (I) as dual Fe/I SAC on N-doped C nanomaterial (Fe/I-N-CR) were successfully synthesized using a metal–organic framework (MOF) as a precursor through a multiple-step synthesis approach (Fig. 6a) [51]. It was observed that the inclusion of I adjusts the electronic structure of the Fe-Nₓ active sites via long-range electron delocalization effects (Fig. 6b-d). Owing to the synergistic effects of dual Fe/I atoms and the structural advantages of 1D nanorods, the Fe/I-N-CR electrocatalyst demonstrated exceptional ORR performance, outperforming both Pt/C and individual Fe or I SACs, counterparts. The assembled quasi-solid-state ZABs achieved a power density of 197.9 mW cm^−2^ and an impressive stability by maintaining the performance at 20 mA cm^−2^ for 280 h, significantly surpassing commercial Pt/C + IrO₂ (119.1 mW cm^−2^ and 47 h) (Fig. 6e-g). This DAC also proved capable of operating across a wide temperature range from − 40 to 60 °C, highlighting its potential for applications in environments with fluctuating temperatures. The proper selection of a compatible electrolyte demonstrated as a promising strategy to achieve high-stability of Zinc/electrolyte and hence the overall superior cycling stability of the Fe/I-N-CR-based ZAB.

Furthermore, the triple metal SACs were reported to boost the bifunctional catalytic performance for MABs compared to SACs and DACs [66–68]. The mechanism of oxygen evolution and reduction reactions involves multiple electron transfer processes, and the synergistic effects of multiple metals are known to advance the performance of these oxygen reactions. Hence, in these dual and triple SACs, the synergy effect between atomic single sites is cited to boost both the ORR and OER catalytic performance and the overall performance for MABs. For example, DACs of FeN₄ and NiN₄ were atomically dispersed in nitrogen-doped graphene, leveraging graphene oxide’s ability to anchor metal ions [68]. This study revealed that Fe/Ni–N–C species such as FeN_4_, NiN_4,_ and Fe/Ni–N_4_ synergistically enhanced the bifunctional catalytic activity. The catalyst with an iron-to-nickel ratio of 1/3 (Fe/Ni(1:3)-NG) improved the power density, specific capacity, and durability [68]. This was attributed to DACs and triple-atom catalysts having a higher metal loading and adaptable active sites that can modulate the d-band center through interactions between electron orbitals, thus adjusting the adsorption energy of intermediates [69].

It is very challenging to demonstrate the distinction function and at which level the active sites in composite materials such as the coexisting active sites of FeN_4_, NiN_4,_ and Fe/Ni–N_4_ in Fe/Ni(1:3)-NG for bifunctional OER and ORR [68]. Ma T. et al. demonstrated the specific function of active sites involved in a Janus DACs embedded in hollow graphene nanosphere composites (Ni-N_4_/GHSs/Fe-N_4_) comprised of Ni-N_4_ and Fe-N_4_ sites [70]. As demonstrated by both experimental characterization and density functional theory (DFT) calculations, the functionality was associated with the microenvironments interfaces of the graphene hollow nanospheres (GHSs) with the active sites, where the outer Fe-N_4_ clusters primarily contribute to ORR, while the inner Ni-N_4_ clusters were responsible for driving the OER. Hence, it is evident that further investigations are needed to understand the synergistic effect, particularly whether they occur to the same extent for OER and ORR in bifunctional catalysts. For such composite materials, it remains unclear whether some active sites dominate one reaction over the other, or if some are even inactive, yet still contribute to an overall enhancement in bifunctional catalytic activity. This further highlights a promising research direction: the preparation of composite materials with distinct active sites for each specific reaction, which balances the competition of both reactions and could minimize active site degradation caused by differing requirements and the unavoidable transformations that occur when both reactions take place at the same active site.

**Fig. 7:**
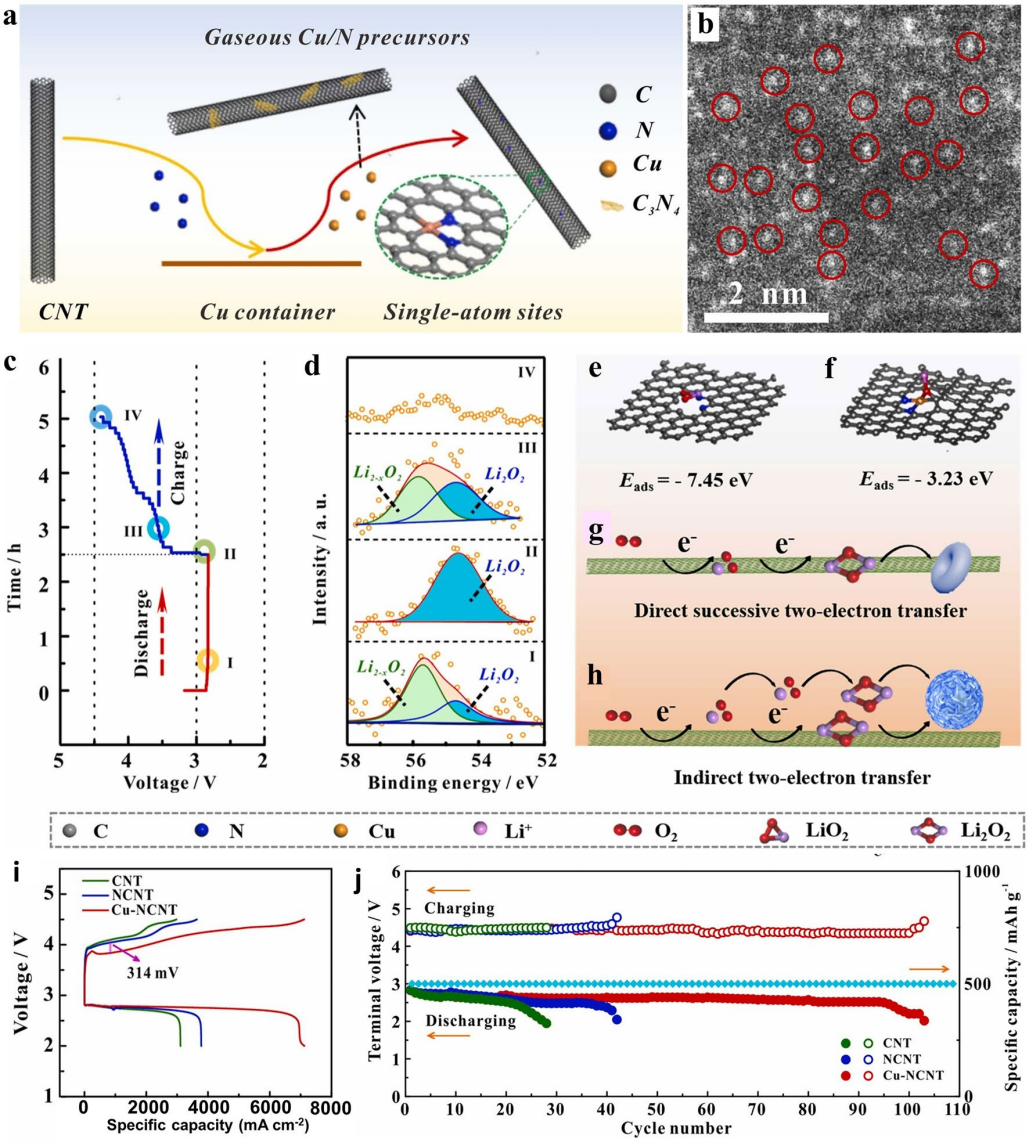
**a** Schematic depicting the Cu-NCNT preparation process. **b** Spherical aberration-corrected HAAFD-STEM image. **c** Schematic diagram depicting the stages of LiO_2_ intermediate on a curves of discharge/charge at 200 mA g^−1^, and **d** XPS spectrum of Li 1*s* correlated with the stages (I, II, III, and IV) shown in Fig. 7c. **e**,** f** Schematic illustration of structures and binding energy of intermediate LiO_2_ on the **e** NCNT and **f** Cu-NCNT. **g**, **h** Scheme depicting the pathways for the formation of the Li_2_O_2_ on the **g** NCNT and **h** Cu-NCNT electrocatalysts. **i** Comparative galvanostatic discharge/charge curves at 200 mA g^−1^ in a voltage range of 2.0 and 4.5 V. **j** Stability performance of LAB cell. Reproduced with permission [71]. Copyright 2022, Elsevier

Owing to the advantages of the high dispersibility of SACs, they offer superior atomic utilization and unsaturated coordination at the active center, significantly enhancing catalyst activity. SACs based on earth-crust rich metals such as Fe, Cu, and Co have also been explored for LAB [61]. Recently, Li et al. designed and constructed CuN_2_C_2_ SACs electrocatalyst for potential application as a catalyst layer in lithium-oxygen batteries (Fig. 7a) [71]. These CuN_2_C_2_ SACs were successfully distributed on the carbon nanotube (CNT) matrix via a confined self-initiated dispersing strategy, as represented in Fig. 7b*.* To understand the reaction mechanism of lithium-oxygen battery operation using the as-prepared CuN_2_C_2_ SACs, the structural evolution of Li_2_O_2_ was found to correlate with the discharge/charge stages I–IV (Fig. 7c-h). It was demonstrated that Li_2−x_O_2_-intermediate formed on the cathode surface during discharge through an indirect pathway (O_2_ → LiO_2_ → Li_2_O_2_) and reappeared during recharging. This showed the reversible formation and decomposition of Li_2_O_2_, facilitated by CuN_2_C_2_ moieties, thus providing better long-term performance. It was revealed that the CuN_2_C_2_ as active sites played a crucial role in shaping the distribution, structure, and catalytic mechanism of Li_2_O_2_, as revealed by DFT calculations. When the CuN_2_C_2_ electrocatalyst was used as a bifunctional material at the cathode, the LOB exhibited superior performance with a discharge specific capacity of 7122 mAh g^−1^ at 200 mA g^−1^, significantly outperforming batteries with CNT (3782 mAh g^−1^) and NCNT (3109 mAh g^−1^) catalysts counterparts (Fig. 7g, h). As the SACs field is a new research direction, further studies are required for understanding the degradation pathways and catalytic mechanisms of SACs bifunctional ECs for MABs.

Although there have been tremendous breakthroughs in the development of techniques enabling reproducible production, such as the self-assembly technique, to the synthesis of a precursor followed by post-treatment, bridging the trade-off between activity and stability is critical and requires more effort. Additionally, the atomic microenvironment modulation of the single metal site by introducing the neighboring metal clusters or by introducing extra heteroatoms (B, P, S, I) has proven to be crucial in boosting the performance and enhancing the long-term stability [51, 52]. Another challenge is that these SACs are supported on a carbon framework, which might undergo degradation in oxidative conduction of oxygen electrocatalysis [72]. This degradation severely affects the SACs and results in their dissolution or aggregation, leading to a drop in performance. Although the techniques developed for stabilizing carbon nanomaterials in the conduction can be transferable to the SAC for oxygen electrocatalysis, some of them face challenges, for example, the high graphitization degree, which increases the corrosion, and stability is associated with a low content of defects, which reduces the intrinsic activity of catalysis. Hence, novel approaches to developing stable SACs are crucial, such as suppressing harmful defects in graphitized carbon or employing alternative supports such as inorganic materials or emerging two-dimensional materials such as MXene.

### Spinels

**Fig. 8:**
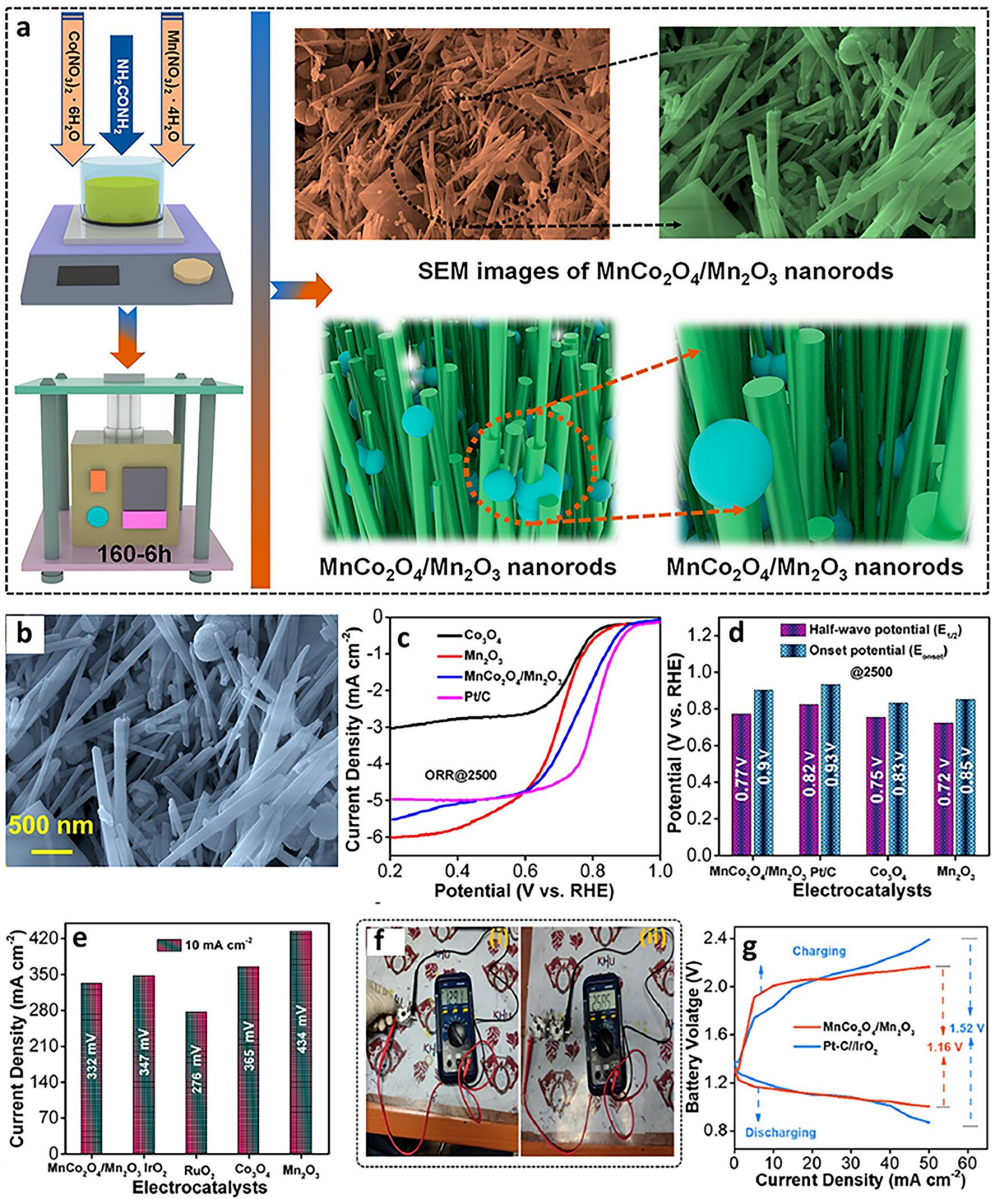
**a** Diagram of the preparation method for MnCo_2_O_4_/Mn_2_O_3_ nanorods (NR) catalyst. **b** High-magnification FE-SEM images of the MnCo_2_O_4_/Mn_2_O_3_ NR, **c **ORR curves of the MnCo_2_O_4_/Mn_2_O_3_ NR, Mn_2_O_3_, Co_3_O_4_, and Pt/C catalysts at a rotation of 2500 rpm. **d** Corresponding onset and half-wave potentials. **e** OER curves of materials in Fig. 8c. **f** OCP of ZAB: (i) single battery and (ii) series-connected two batteries. **g** Comparison of charging and discharging curves of the MnCo_2_O_4_/Mn_2_O_3_ NR-based ZAB and Pt/C//IrO_2_-based ZAB. Reproduced with permission [77]. Copyright 2024, American Chemical Society

Spinel metal oxide materials stand out as promising catalysts for oxygen electrocatalysis due to their structural stability and flexibility for developing double and triple transition metals for advanced activity due to the synergy between the metal elements [73, 74]. In MABs, mono-metal spinel oxides such as manganese and cobalt have shown promising performance stability as promising alternatives to PGM-free catalysts, especially for ORR due to their excellent properties and considerably low cost [75, 76]. It was further observed that the introduction of transition metals such as Co in manganese oxide catalysts boosts the bifunctionality of manganese-based catalysts and has potential applications in MABs. For example, manganese cobalt oxide/manganese oxide (MnCo_2_O_4_/Mn_2_O_3_) nanorod (NR) materials synthesized using a facile, reproducible, and scalable one-step hydrothermal technique without calcination showed improved OER and ORR (Fig. 8a, b) [77]. This approach enabled the synthesis of a hybrid architecture composed of nanorods and nanospheres, which are crucial for enhancing surface area and diffusion pathways for the transport of electrons and electrolyte ions during electrocatalytic applications. The observed improved oxygen electrocatalysis bifunctional performance of MnCo_2_O_4_/Mn_2_O_3_ NR compared to Mn_2_O_3_, as shown in Fig. 8b-d, was due to the inclusion of cobalt oxide in manganese oxide, which resulted in better kinetics for both reactions. Although this catalyst exhibited better bifunctional activity considering the ΔE, its OER and ORR were less performing compared to IrO_2_ and Pt/C, respectively, requiring further improvement. Despite that, the evaluated voltage gaps of ZAB using MnCo_2_O_4_/Mn_2_O_3_ NR and Pt/C//IrO_2_ electrocatalytic materials at the cathode were 1.16 and 1.52 V, respectively (Fig. 8e). The fabricated ZAB using this optimized catalyst showed a low potential voltage at high current densities and better durability over 68 cycles (∼20.3 h) compared to the ZAB fabricated using Pt/C//IrO_2_ electrocatalysts (Fig. 8e, f). Furthermore, the trimetallic spinel-types, such as Co_0.5_Ni_0.5_Mn_2_O_4_ showed higher bifunctional electrocatalytic activity toward ORR and OER and better cathodic performance for MABs than bimetallic spinel-type oxides such as CoMn_2_O_4_ and NiMn_2_O_4_ [78]. As discussed, OER and ORR show different mechanisms, and using *in situ* X-ray absorption spectroscopy, the trimetallic spinel-type revealed that the Co was the driving force for ORR process than the magnesium and nickel as observed from the oxidation state shift.

**Fig. 9:**
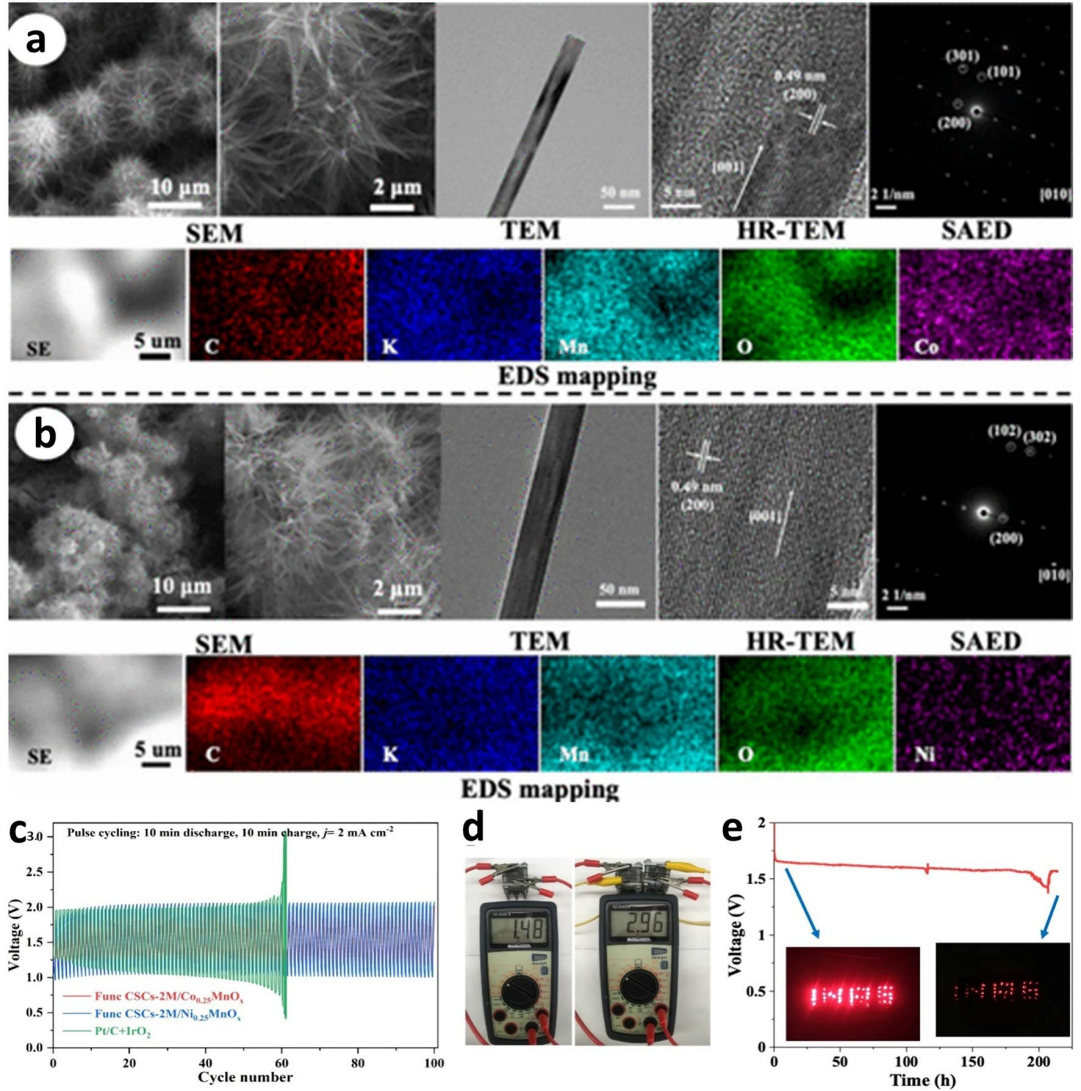
**a, b** Physical and chemical characterizations of Func CSCs-2 M/Co_0.25_MnO_x_ (Panel a) and Func CSCs-2 M/Ni_0.25_MnO_x_ (Panel b). **c** GCD cycling curves at 2 mA cm^–2^ when the Func CSCs-2 M/Co_0.25_MnO_x_ cathode is used to construct a ZAB, **d** OCVs of a single and two ZABs connected in series, and **e** pictogram of voltage over time for two-series-connected ZABs featuring the INRS logo. Reprinted with permission [80] . Copyright 2024, American Chemical Society

Although manganese oxides (MnO_x_) are promising ORR catalysts, their intrinsic low conductivity limits their bifunctional application as they exhibit poor OER [79]. However, through the incorporation of heteroatoms such as carbon and nitrogen, improved bifunctional performance for MAB was demonstrated. Furthermore, doping metal elements to these composite materials of MnO_x_ and carbon nanomaterials further enhances the performance [79]. Recently, it was reported that the doping of Co or Ni in a self-supported α-MnO_2_ nanorod arrays on surface hierarchical carbon sphere (Func CSCs-2 M/Co_0.25_ (or Ni_0.25_) MnO_*x*_) using a reproducible hydrothermal (HT) approach proved by the synthesis of a series of Func CSCs-2 M/Ni_*y*_MnO_*x*_ (Fig. 9a, b) [80]. This catalyst exhibited outstanding trifunctional performance for MAB and for water electrolysis compared to the pristine Func CSCs-2 M/MnO_2_ electrocatalysts (Fig. 9c). Two ZABs, connected in series, fabricated using electrochemically functionalized cathodes, powered red light-emitting diodes over 200 h (Fig. 9d, e), showing outstanding stability. The improved performance compared to the previously reported Func CSCs-2 M/MnO_2_ electrocatalysts was not only attributed to the incorporated functionalized hybrid Func CSCs with impressive conductivity [81], but mainly to the synergistic enhancement of the ORR/OER catalytic activity due to Co or Ni dopants in α-MnO_2_. This shows a unique strategy to improve the bifunctional performance through the synthesis of hybrid composites of α-MnO_2_ materials, which can be extended to other materials.

The bifunctional catalytic activity of cobalt-based spinels oxide was found to be improved through combining multiple elements with cobalt-spinel. Using this strategy, the inclusion of lithium (Li) by chemically delithiation of cobalt oxide to form LT-Li_1−x_CoO_2_ showed improved ORR and OER activities better than Co_3_O_4_, making it a highly performing bifunctional material for MABs [82]. Additionally, doping manganese in nanoparticles grown on carbon nanotubes (Mn-Co_3_O_4_@CNTs) showed improved bifunctional catalytic performance for both OER and ORR and, thus, potential catalysts for MABs [75].

Furthermore, the replacement of oxygen in spinel catalysts with other non-metal elements has shown enhanced bifunctional catalytic activity toward MABs. The replacement of oxygen by such non-metal elements like sulfur to form metal sulfides offers better electrical conductivity compared to metal oxides [11]. To boost the OER and ORR catalytic activity, the effect of doping transition metals such as Ag, Fe, Mn, Cr, V, and Ti in cobalt–nickel sulfide spinels using a continuous hydrothermal flow synthesis (CHFS) method was evaluated [83]. The Mn (III) cation doping was found to induce a desirable electronic structure in the active sites of Mn-doped cobalt–nickel sulfides. As a result, a ZAB showed a power density of 75 mW cm^−2^ at a *j* of 140 mA cm^−2^, which was a 12% increase in the power density compared to the undoped control sample.

### Perovskites

Perovskite oxides, which are characterized by an ABO_3_ structure, where A and B stand for an alkali- or rare-earth metal and a transition metal, respectively, are competent bifunctional oxygen electrocatalysis catalysts owing to their excellent stability and adjustable electrochemical properties [47]. Their catalytic performance is governed by the characteristics of surface cations, which are affected by oxygen deficiencies and structural mutations from the fundamental cubic crystal structure [84]. The catalytic activity in B-octahedral cations in perovskite-type oxide is influenced by the metal–oxygen electronic states [85]. Specifically, π-bonding and π^*^-antibonding orbitals form through the hybridization of transition metal d orbitals with oxygen’s 2*p* orbitals, where the overlap strength of these orbitals determines the interaction with oxygen species. Shao-Horn and colleagues proposed that the filling of the e_g_ orbital on the surface B-site cations serves as a key descriptor for the catalytic efficiency in perovskite oxides [86].

**Fig. 10:**
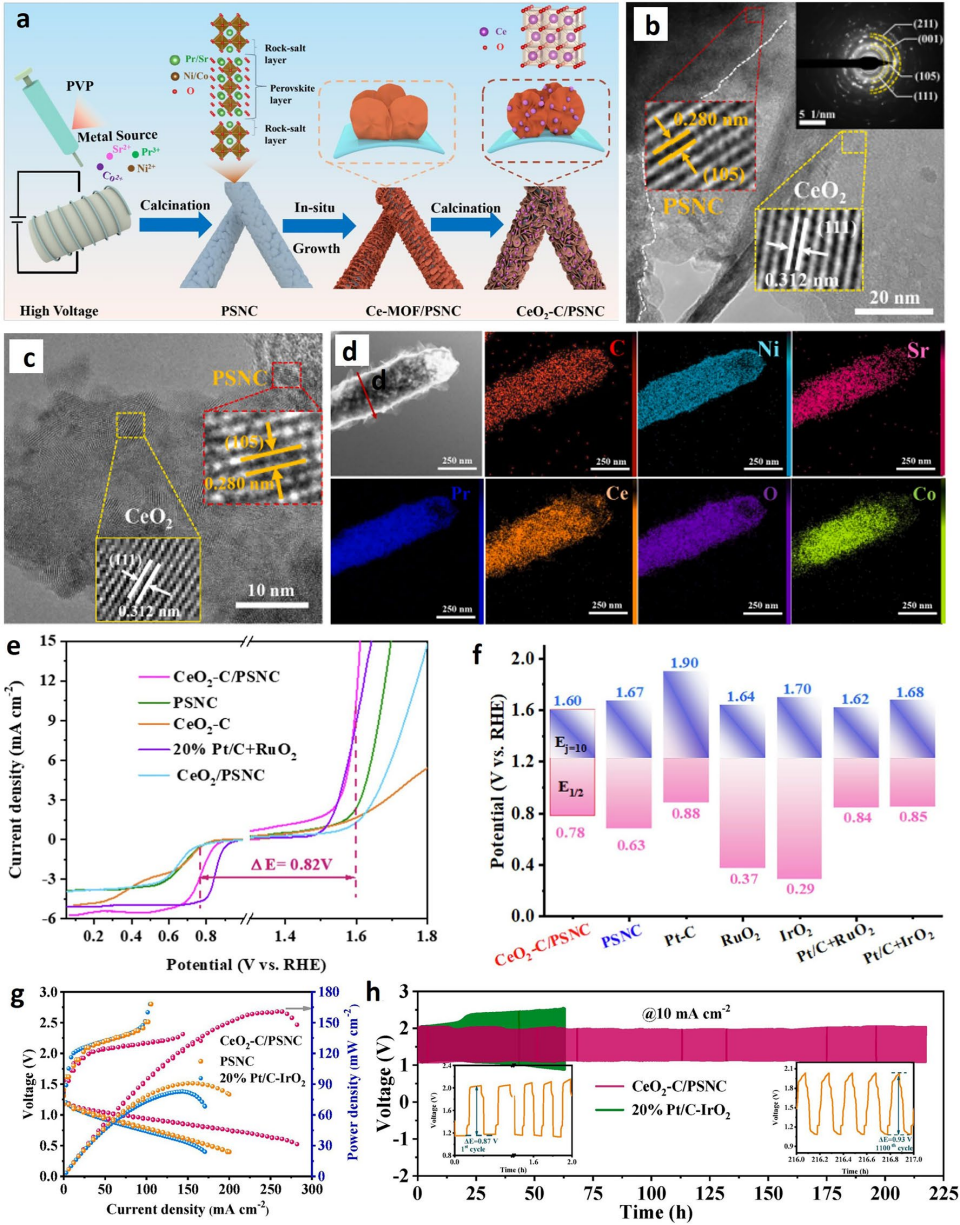
**a** Schematic representation for the synthesis of CeO_2_-C/PSNC catalyst. **b, c** HRTEM images. **d** TEM-EDS mapping, **e** LSV curves for bifunctional characterization, with an error estimation of ± 2 m V. **f** Polarization curves of charge and discharge, as well as of power density plots of the ZABs constructed with 20% Pt/C-IrO_2_, CeO_2_-C/PSNC, and PSNC as catalyst on cathode. **g** Bar diagram of voltage gap between E_1/2_ for ORR and E_j=10_ for OER with selected highly efficient catalysts. **h** Cycling curves investigating the stability. Reproduced with permission [87]. Copyright 2024, Elsevier

Deng et al. reported a perovskite/CeO_2_/carbon heterojunction (CeO_2_-Pr_3_Sr(Ni_0.5_Co_0.5_)_3_O_10−δ_ nanofibers (CeO_2_-C/PSNC), which was prepared by a multistep engineering strategy comprising electrospinning, microwave in situ growth, and calcination treatment (Fig. 10a) [87]. The microscopical analysis reveals the tight inclusion of CeO_2_ in the porous of PSNC nanofibers, modifying its microenvironment properties (Fig. 10b, c). Figure 10d shows a homogeneous distribution of element constituents, which indicates that the synthesis approach was successful. The half-wave potential of this Ruddlesden–Popper perovskite composite for ORR was 0.78 V, while an *η* required to afford a current density (*j*) of 10 mA cm^−2^ was 370 mV (Fig. 10e, f). The hierarchical nanostructure, strong electron interaction, oxygen vacancies, and abundant active sites were attributed to be the origin of advanced bifunctional ORR/OER performance in alkaline solution. When this electrocatalyst was used as a bifunctional ORR/OER catalytically active material on the air cathode, the assembled ZAB showed excellent performance with a power density of 161 mW cm^−2^ (Fig. 10g) and a promising cycling life of over 219 h (Fig. 10h). Furthermore, this electrocatalyst enabled the fabrication of all-solid-state ZAB with an OCV of ∼1.44 V, good flexibility, and durability. Although the 20% Pt/C + RuO_2_ catalysts exhibited roughly similar ΔE of 0.83 V compared to CeO_2_-C/PSNC (0.82 V), there is a clear trade-off between the activity and the stability when comparing these two catalysts. The ZAB constructed using 20% Pt/C + RuO_2_ catalysts only lasted around 65 h, as shown in Fig. 10i, less than a third of the CeO_2_-C/PSNC. This demonstrates that the trade-off between activity and stability should be considered as a crucial parameter for practical applications, rather than relying on the performance of half-self-chemical reactions.

The substitution of guest metal in the perovskites such structure has been reported to the ORR and OER activities. For instance, the proper substitution of cobalt by molybdenum at a certain ratio in LaCoO_3_ (LCO) was reported to improve the ORR/OER bifunctional catalytic activity [88]. The optimized Mo-doped LaCoO_3_ (LCO) at a Co: Mo ratio of 95: 5 (LaCo_0.95_Mo_0.05_O_3_ (LCM-5)) reached a *j* of − 0.1 mA cm^−2^ for ORR at 0.861 V, while a reduced *η* of 405 mV was required to reach a *j* of 10 mA cm^−2^ for OER. Furthermore, when the optimized LCM-5 was used as a cathode in a ZAB, a power density of 136.1 mW cm^−2^ and a specific discharge capacity of over 800 mAh g^−1^ was reported. Additionally, this ZAB showed a long-term cycle capability to maintain 10 mA cm^−2^ for 120 h. This excellent catalytic activity was attributed to the doped Mo, which regulated the B-site Co valence states and increased the surface oxygen vacancies [88].

Furthermore, the synthesis of hybrid complex material of perovskite oxide and carbon nanomaterials was found to be a successful strategy to tailor the bifunctional electrocatalytic activity of perovskite oxide [89]. For example, a hybrid of cobalt-doped lanthanum manganese oxide (LaMnO_3_) and N-doped C nanotube (LMCO/NCNT) was developed by using the LaMnO_3_ perovskite as a growth substrate for NCNT and showed bifunctional performance [89]. This LMCO/NCNT hybrid demonstrates significant ORR enhancement with an onset of − 0.11 vs. SCE and half-wave potentials of − 0.24 V vs. SCE. It further required 0.9 V vs. SCE to reach a *j* of 27 mA cm^− 2^ for OER. Additionally, both of these strategies, metal substitution in perovskites and engineering hybrids with carbon, were proved to be effective in tailoring the bifunctional activity of perovskites for LABs. Hsu et al. investigated the effect and appropriate ratio of La by forming a series of La₁₋ₓSrₓCoO_3-δ_ (x = 0.1, 0.3, and 0.5). Due to the rise in the number of oxygen vacancies and surface area, the La₀.₅Sr₀.₅CoO_3-δ_/Super P carbon cathode showed the highest discharge capacity (6,032 mAh g^−1^) for LAB [90]. For additional bifunctional perovskite catalysts, refer to Tables 1 and 2 for ZABs and LABs, respectively.

**Table 1:** Recent earth-abundant bifunctional perovskite electrocatalysts for ZABs

Electrocatalysts	Load (mg cm^-2^)	Electrolyte	ORR E_1/2_ (V vs. RHE)	OER E_j = 10_ (V vs. RHE)	ΔE (E_j = 10-_E_1/2_)	References
Commercial Pt/C-IrO_2_	0.1	0.1 M KOH	0.86	1.93 (Pt/C)/1.60	1.07/0.76	[91]
Commercial Pt/C-IrO_2_ (1:1)	0.226	0.1 M KOH	–	–	0.83	[87]
CeO_2_-C/PSNC	0.226	0.1 M KOH	0.78 V	1.6	0.82	[87]
LaCoO_3_	0.189	0.1 M KOH	0.518	1.812	1.294	[88]
La_0.85_Ba_0.15_CoO_3_	0.189	0.1 M KOH	0.536	1.685	1.149	[92]
LaCo_1–x_Mo_x_O_3_ (x = 0.05)	0.189	0.1 M KOH	0.595	1.705	1.11	[88]
La_0.95_FeO_3-δ_	0.232	0.1 M KOH	0.56	1.64	1.08	[93]
P doped LaFeO_3_	0.255	0.1 M KOH	0.66	1.69	1.03	[94]
La_0.8_Sr_0.2_Co_0.4_Mn_0.6_O_3_	0.255	0.1 M KOH	0.686	1.736	1.05	[95]
LaMnO_3_**₋**CoO	0.232	0.1 M KOH	0.56	1.78	1.22	[96]
La_0.7_Sr_0.3_MnO_3_/Fe-1.5	1.0	0.1 M KOH	0.777	–	0.942	[97]
Ce_0.9_Gd_0.1_O_2-δ_ (GDC) decorated (Pr_0.5_Ba_0.5_)CoO_3-δ_ (PBC)	–	0.1 M KOH	0.56 vs. RHE (E_ORR_ (V) @-1 mA cm^−2^)	1.69	1.13	[98]
Ba_0·5_Sr_0·5_Co_0·8_Fe_0·2_O_3_/NCNT	0.510	0.1 M KOH	0.86	1.62	0.76	[99]
La(Fe_0.2_Co_0.3_Mn_0.1_Cr_0.2_Zn_0.2_)O_3–δ_	–	0.1 M KOH	0.41	1.526	1.042	[100]
La_0.75_Sr_0.25_Mn_0.5_Fe_0.5_O_3_	1.0	0.1 M KOH	0.721	1.658	0.94	[101]
nsLaNiO_3_/NC	–	0.1 M KOH	0.64	1.66	1.02	[102]

**Table 2:** Recent earth-abundant bifunctional perovskite electrocatalysts for LABs

Electrocatalysts	Specific capacity (mAh g^ − 1^ @mA g^ − 1^)	Overpotential (V@mAh g^ − 1^@mA g^ − 1^)	Cycle life (cycles@mAh g^ − 1^@mA g^ − 1^)	References
Pt-HCNPs	16000	0.41@100	40	[103]
RuO_2_/CNTs	1000	0.6@200	50	[104]
α-Fe_2_O_3_-LaFeO_3–*x*_	7183@100	1.0@500@100	108@500@100	[105]
Ag@La_0.6_Sr_0.4_Fe_0.9_Mn_0.1_O_3_	12477	1.3@500@400	147@500@400	[106]
Ni-La_0.9_Mn_0.6_Ni_0.4_O_3–*δ*_	16,656@400	–	95@500@400	[107]
La_0.8_Sr_0.2_Mn_0.6_Ni_0.4_O_3_	5364@50	1.074 (ΔE(V))	79 cycles@ 500 mAh gcarbon^–1^	[108]
Sr-doped La_2_NiO_4_/NiO	131380@200	0.66@500@500	188@500@200	[109]
LaNi-_0.5_Co_0.5_O_3_	7.96 mAh cm^ − 2^@0.1 mA cm^ − 2^	0.05@0.5 mA cm^−2^@0.1 mA cm^−2^	100@0.5 mA cm^−2^@0.1 mA cm^−2^	[110]
La_0.5_Sr_0.5_CoO_3–*x*_ (HPNLSC/KB)	5799	1.14 V@0.025 mA cm^−2^	50@500	[111]
La_0.8_Fe_0.9_Co_0.1_O_3–*δ*_	7270.1@100	0.7 V@500@200	215@500@500	[112]
Ni_3_S_2_/PrBa_0.5_Sr_0.5_Co_2_O_5 + *δ*_	12874@100	0.68@1000@100	120@1000@100	[113]
La_0.7_MnO_3–*δ*_	29286@50	0.38@1000@200	375@1000@300	[114]
Fe_2_O_3_/LaNiO_3_	10419@100	0.77 V@50 mA g^−1^	90@500	[115]
S-doped LiNaO_3_	24067@100	0.37@1000@200	347@1000@100	[116]
LaF_3_/La_0.8_Fe_0.9_Co_0.1_O_3–*δ*_	7373.5@100	1.29@500@200	157@500@200	[117]

### Metal-Free Carbon-Based Bifunctional Catalysts

Carbon-based materials have received great attention for numerous catalytic applications, both as catalyst support or as directly active material, such as in metal-free carbon materials. This is mainly due to their superior electrical conductivity, high surface area, and tunable chemical properties [118]. Furthermore, the raw materials for the synthesis of carbon nanomaterials are abundant, such as biomass, making them low cost, and thus a viable-economy materials for large-scale applications [119]. In addition to their low cost, their chemical stability and ability to be functionalized with heteroatoms or metals make them versatile and efficient for applications such as water electrolyzers, fuel cells, batteries, and supercapacitors. As the search for bifunctional materials for MABs is crucial, the heteroatom doping in commercial carbon nanomaterials and biomass-derived carbon materials can be employed to enhance their catalytic performance by modifying the electronic structure and surface chemistry, thereby enabling desirable properties for bifunctional OER and ORR [120]. Doping with non-metal elements like nitrogen, sulfur, or phosphorus introduces active sites, improves charge transfer, and alters the adsorption energy of intermediates [121], thereby boosting catalytic activity and selectivity for reactions such as the ORR and OER without the addition of metal elements.

To address a major concern of carbon corrosion at high potentials during oxygen reactions such as OER, a systematic corrosion mechanism of carbon study was conducted to support the possibility of using carbon-free metal in MABs [122]. This was done by investigating the correlation between the structural properties of carbon, graphitization, surface, and the electrochemical catalytic activity of carbon black, CNTs, and graphene as catalyst carriers. It was found that the CNTs characterized by high crystallinity and less edge exposure showed superior performance over activated carbon black and graphene for MABs. Hence, it is critical to develop bifunctional electrocatalysts based on carbon nanomaterials, but also considering this challenge of carbon corrosion, especially in highly alkaline conditions of practical MABs. Hence, it is critical to develop bifunctional electrocatalysts based on carbon nanomaterials but also considering this challenge of carbon corrosion, especially in highly alkaline conditions of practical MABs. Zhao et al., developed dual-doped and metal-free porous carbon materials using the pyrolysis of MOF containing Zn, N, and B as a precursor [123]. The doped-heteroatoms (B and N) were homogeneously distributed in carbon material, and the optimized BNPC-1100 material demonstrated a bifunctional OER and ORR due to B-N dual-doping, high porosity, and high pyridinic N content. When used as an air-breathing electrode in a ZAB, a discharging voltage was 1.12 V and showed excellent stability up to 100 h without severe deviation from the initial voltage.

Furthermore, phosphorus is another heteroatoms that boost the bifunctionality activity toward oxygen electrochemical reactions and even for the hydrogen evolution reaction [124]. Researchers developed a scalable method to create nitrogen and phosphorus-codoped carbon nanospheres (NPCs) with cesium chloride assistance, leaving no residual metals after acid treatment. This metal-free electrocatalyst shows strong bifunctional catalytic activity, with an ORR half-wave potential of 0.85 V and a *η* of 0.34 V to achieve 10 mA cm^−2^ for OER. Fabricated ZAB using this catalyst performs comparably or even better than Pt/C or IrO₂ catalysts [125]. The strategy of doping heteroatoms in carbon-based nanomaterials yields tailored bifunctional catalytic activity for OER and ORR and shows practical performance in LAB. Elumalai et al. developed a strategy involving the carbonization of paper cups followed by chemical activation to further dope carbon with Selenium, Nitrogen, and Boron [126]. The Se heteroatom-doped carbon (SeC900) excelled in the bifunctional OER and ORR performance due to its high surface area, which facilitated good O_2_ adsorption. The LAB coin cell, where the SeC900 was used as an active catalyst on the air-breathing electrode, exhibited an OCV of 3.14 V and an excellent discharge capacity of 1618 mAh g^−1^ @50 A g^−1^.

### Metal–Nitrogen-Doped Carbon-Based Materials

To further boost the bifunctional catalytic activity of carbon materials, the synthesis of transition metal interacting with nitrogen-doped carbon (metal–nitrogen–carbon) nanomaterials is an effective approach [127]. For example, a Fe–N/C material demonstrated superior performance to the well-agreed a-MnO_2_ in the Li–air cell [128]. This advanced performance was due to the dissolved iron phthalocyanine (FePc) in organic electrolyte, which served as a shuttle of (O_2_)^−^ species and electrons between the electronic conductor and the insulator Li_2_O_2_. It also blocked any possibility of forming Li_2_O_2_ product and decomposed afterward to directly interact with carbon, ensuring better stability. As biomass-derived materials are abundant, Liu et al., prepared defect-abundance carbon sheets derived from biomass with N doping (GPNCS) from fruits of glossy privet using a hydrothermal-activation-N-doped strategy, and this material showed promising ZAB [125]. The advanced activity was attributed to synergism between N-doping atoms and topological defects. When the GPNCS was used as a cathode in a ZAB, a low charge–discharge voltage gap was demonstrated, and at a *j* of 10 mA cm^–2^, it demonstrated a promising durability up to 1340 cycles (about 500 h).

**Fig. 11:**
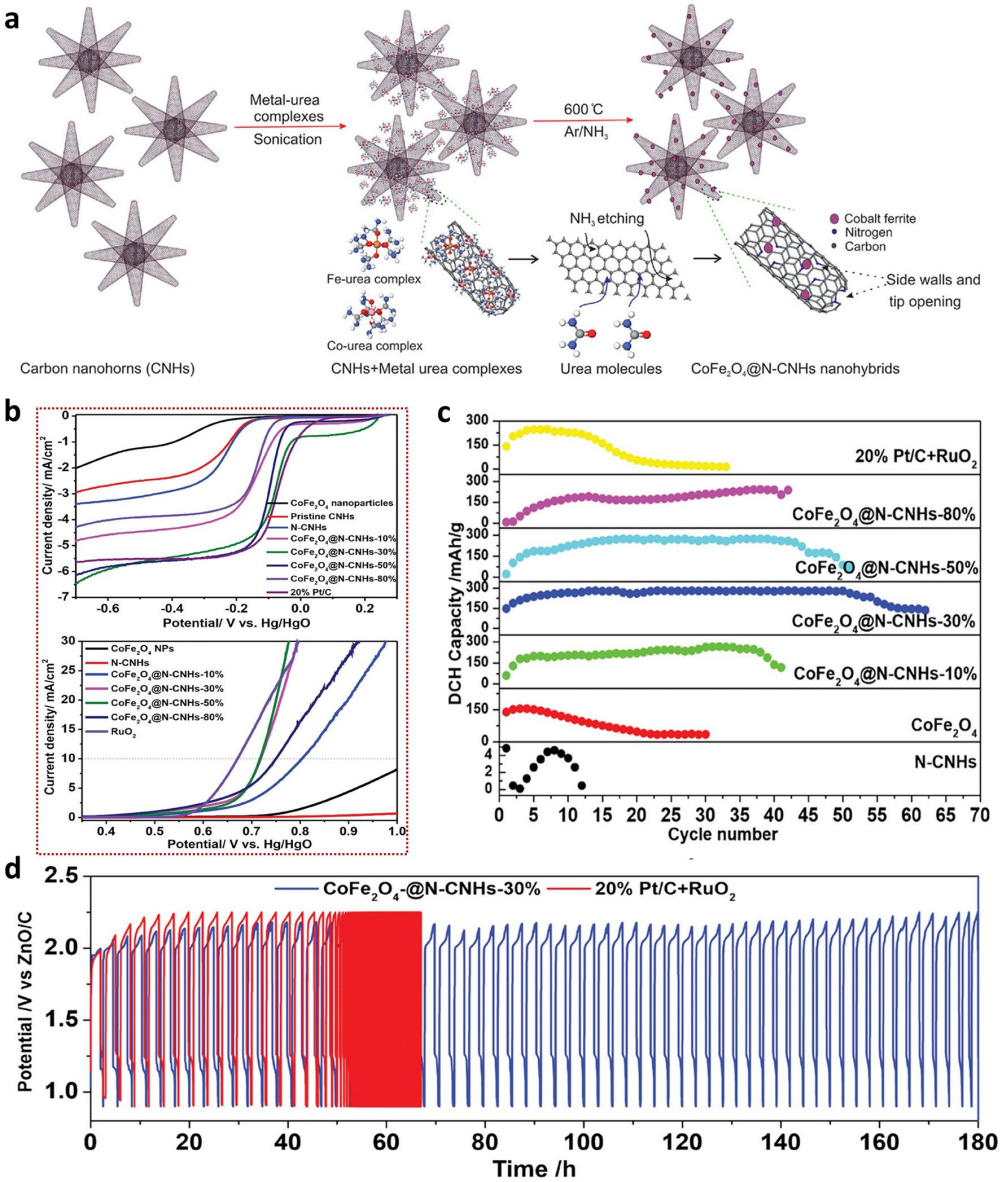
**a** Schematic representation simplifying the fabrication of CoFe_2_O_4_@N-CNHs. **b** LSV curves for ORR and OER. **c** Plot of specific discharge capacity vs. cycle number, **d** charge/discharge cyclization curves at the j of 0.55 mA cm^−2^ with 3 h per cycle. Reproduced with permission [129]. Copyright 2024, John Wiley and Sons

Additionally, the synthesis of a hybrid of metal nanomaterials with carbon enables excellent catalytic properties. As most N-doped carbon nanomaterials show outstanding ORR activity but relatively poor OER performance, combining a carbon-nanomaterial with a highly OER-performing material, such as nickel iron oxides, resulted in outstanding performance [129]. This tailored bifunctional catalytic activity was prepared by using a straightforward one-step annealing method of cobalt and iron precursors with carbon nanohorns under an Ar/NH₃ atmosphere (Fig. 11a). The resulting cobalt ferrite@N-doped C nanohorns (CoFe_2_O_4_@N-CNHs) showed ORR potentials comparable to standard Pt/C catalysts and outperformed RuO_2_ catalysts in OER efficiency (Fig. 11b). Additionally, ZABs with this nanohybrid catalyst exhibit enhanced cycle stability and energy density compared to conventional Pt/C-RuO₂ systems (Fig. 11c, d). In addition to the outstanding performance, the hybrid CoFe_2_O_4_@N-CNHs-30% catalyst showed better stability compared to pristine N-CNHs and CoFe_2_O_4_. Furthermore, combining different types of carbon nanomaterials, such as nitrogen-doped carbon (NC) and multi-walled carbon nanotubes (MWCNTs) embedding Co/Zn nanoparticles (Co/Zn@NC@MWCNTs), shows an improved ORR and OER bifunctional catalytic activity and ZAB as well [130].

Metal elements in carbon-derived materials play a crucial role in enhancing the bifunctional catalytic activity for ORR and OER. For example, different metal elements were reported to influence the nanostructure and morphology of cobalt-tin sulfide nanopores (CoSnOH/S@C NPs) with a carbon layer prepared by using a two-step hydrothermal procedure [131]. The optimized CoSnOH/S@C NPs exhibit a porous structure with high surface area and high interactions between the CoSnOH/S NPs and carbon layer, leading to superior catalytic performance for ORR with a half-wave potential of − 0.88 V and lower overpotential (*η*) of 429 mV vs. RHE at a *j* of 50 mA cm^−2^ for OER. The resulting ZAB shows better cycling stability and enhanced catalytic efficiency compared to the ZAB fabricated using Pt/C and IrO_2_ electrocatalysts. Earth-abundant materials based on complex materials such as metal macrocycles like porphyrins have also been investigated for MAB. One impressive example is the construction of a pyrolysis-free cobalt porphyrins coordination polymer containing Co–N-C bonding with promising trifunctional activity for HER, OER, and ORR [132]. With the oxygen electrocatalysis’s bifunctional properties, the ZAB achieved a specific capacity of 723 mAh g^−1^. The advanced trifunctional performance and stability are attributed to the dicobalt face-to-face porphyrins, ensuring donor–acceptor characteristics with a band gap of 1.87 eV as calculated by the DFT calculation.

Although the metal hydroxides-based materials show relatively poor performance for bifunctional oxygen electrocatalysis, synthesizing hybrid nanomaterials with carbon–nitrogen was investigated to tailor the bifunctional activity. For example, a multiscale construction strategy was used for a hybrid catalyst made of cobalt-porphyrin complex and NiFe layered double hydroxides [133]. This approach aimed to enhance the activity of metal hydroxide in combination with cobalt porphyrin, which possesses high intrinsic molecular-level activity, both serving as the active sites for oxygen electrocatalysis. As a result, the multiscale-designed electrocatalyst demonstrates a small ΔE for OER/ORR of 0.68 V. The integration of this catalyst into the air cathode of ZAB demonstrated a high power density of 185.0 mW cm^−2^. Additionally, this fabricated ZAB exhibited a remarkable durability of up to 2400 cycles when tested at 5.0 mA cm^−2^.

Developing complex composite materials enables the synthesis of robust bifunctional catalysts due to the modulation of microenvironment structures that is unachievable with simple materials. Particularly, this strategy has emerged as a method to prepare materials with various active sites, allowing each reaction to take place at a selected site [70]. Additionally, the synergistic effects of the constituents in these composites cannot be ignored, raising a critical issue of understanding how complex reactions occur, considering the selectivity of each active site for a particular reaction, while also accounting for possible synergistic or even inhibitory effects [134]. Jiang’s group conducted a comprehensive study using experimental and theoretical characterization to unveil the selectivity of active sites involved in specific reactions during the OER and ORR of atomically dispersed Fe-N_4_ sites bridged with MoO_x_ clusters (FeN_4_/MoO_x_) on carbon black. They also investigated the effect of the interaction of catalyst constituents on the active sites [135]. The FeN_4_/MoO_x_ composite showed a Δ*E* of 0.665 V, which outperformed to commercial Pt/C-IrO_2_ catalyst (0.798 V). It maintained the RZAB performance for around 440 h and exhibited a specific capacity of 819 mAh g^−1^, while the commercial catalyst only lasted for 150 h. The study demonstrated that the Fe–Ni interactions negatively affect the FeN_4_ sites accountable for the ORR, while synergistically enhancing the OER performance of MoO_x_ clusters in the FeN_4_/MoO_x_ composites.

### Transition Metal-Derived MOF Materials

**Fig. 12:**
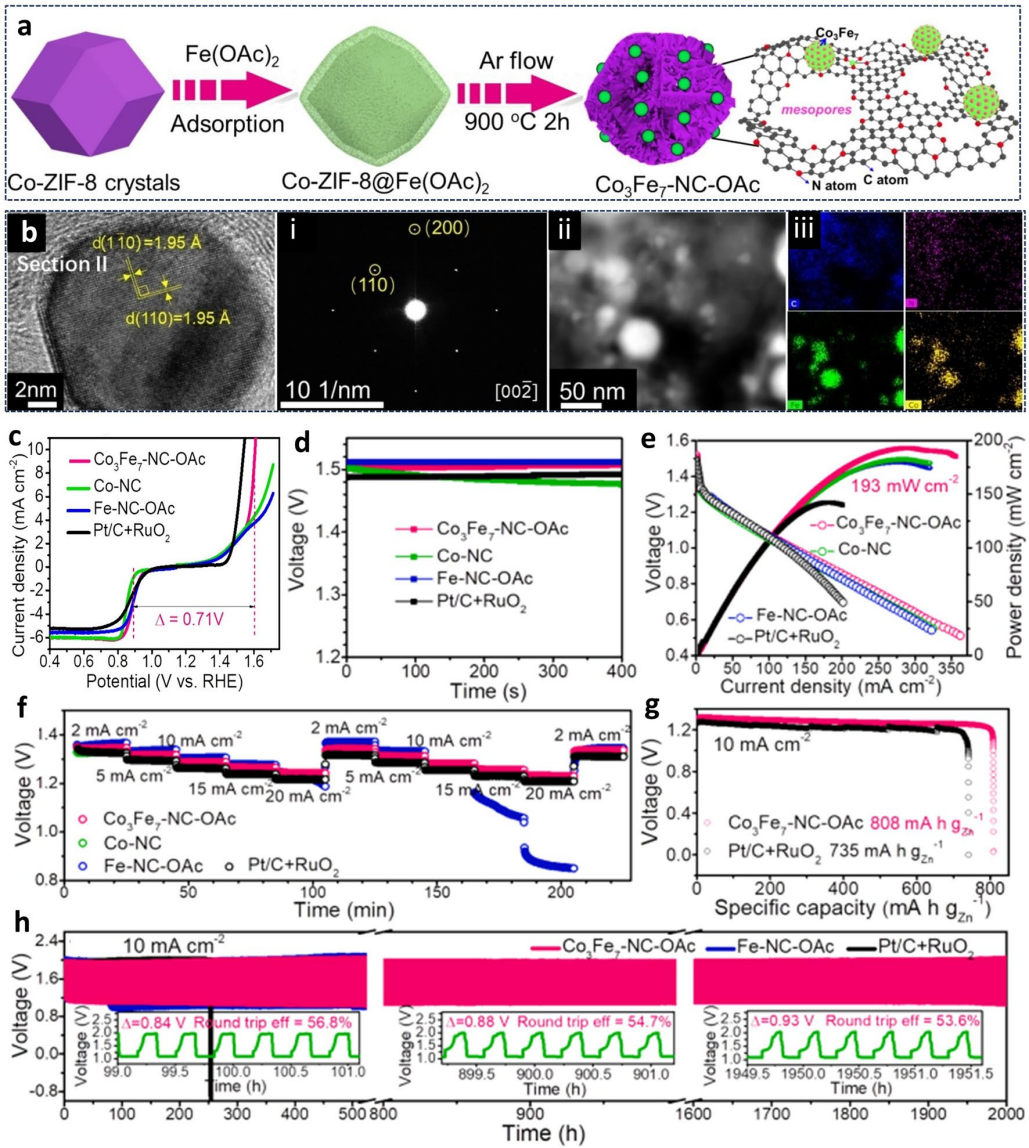
**a** A diagram depicting the synthesis process of Co_3_Fe_7_-NC-OAc, **b** HRTEM image, i) SAED pattern, ii, iii) HAADF-STEM image, and corresponding element mapping image, **c** bifunctional characterization, **d** curves for OCV, **e** polarization curves for discharge and power density plots, **f** comparative discharge curves for ZAB. **g** Specific capacity and **h** galvanostatic cycling tests. The inset depicts the voltage gap and round-trip efficiencies of ZAB at selected times for the constructed battery using Co_3_Fe_7_-NC-OAc. Reproduced with permission [52]. Copyright 2025, Elsevier

MOFs, made by metals or clusters connected by organic ligands, have gained significant attention as multifunctional materials and a versatile platform for synthesizing novel nano-carbon composites [136]. Transition metal-based MOFs are effective O₂ electrode materials, offering uniform pores, high surface area, and tunable chemical environments that enhance O₂ enrichment and transportation within the electrode [137]. Their structural precision, flexibility, and modifiability enable further optimization of battery performance [138]. Metal alloys are promising catalysts for OER and often surpass the performance of corresponding metal oxides in alkaline solution [139]. Anchoring metal alloy-derived catalysts on carbon nanomaterials, such as using MOF as a template, was proposed for tailoring the bifunctional catalytic activity. In this reported strategy, the iron (II) acetate-assisted approach was used to prepare iron-cobalt alloy anchored on carbon nitrides (NC) matrix (Co_3_Fe_7_-NC-OAc) (Fig. 12a(i)) [52]. The alloying of Fe with Co leads to the formation of Co_3_Fe_7_ nanoparticles as ORR/OER active. In addition to the improved activity due to the enlarged pore size to serve as gas transfer channels from the iron (II) acetate incorporation, the synergetic electronic coupling between carbon nitrides matrix and Co_3_Fe_7_ nanoparticles was another feature that enabled enhanced OER and ORR catalytic activity (Fig. 12a(ii)). Superior peak power densities of 587 mW cm^−2^ and 193 mW cm^−2^ in solid-state and aqueous ZABs (Fig. 12b-f), respectively, were achieved using this MOF-derived alloy catalyst as a bifunctional catalyst [52].

The MOF is a promising precursor for synthesizing materials with highly active sites and free of metal aggregation. H. Zheng et al. recently reported a confinement strategy to develop a cobalt carbon nitride (Co@N-CNSs) derived from cobalt(II) phthalocyanine complexes (Co-Pc) immobilized in Zn-MOF followed by the carbonization process [140]. This confinement approach enabled the synthesis of small-sized Co nanoparticles anchored on carbon nitride nanosheets. The ZAB in which the Co@N-CNSs catalyst was used as an active layer on the cathode showed a high specific capacity and a peak power density of 775 mAh gZn^−1^ and 227 mW cm^−2^, respectively. The pyrolysis strategy enables the synthesis of metal composites with carbon nanomaterials using various types of carbon precursors. It was found that pyrolyzed iron phthalocyanine (FePc) with cobalt-doped indium-based MOFs at 800 °C formed FePc@HCoNC with superior activity for ZAB of specific capacity of 758.10 mAh g^−1^, and stable cycling for 150 h [141]. To further boost the performance of metal complex materials, combining with carbon to form composites such as CoPc@CNT composites proved to show improvement in LAB with a discharge capacity of 3400 mAh g^−1^_catalyst_ [142]. MOF-derived materials have emerged as promising candidates for various electrochemical energy technologies. However, while developing robust catalysts, especially for harsh oxidative conditions involved in reactions such as the OER, remains a key challenge, equal effort must also be devoted to addressing issues such as cost-effectiveness, scalability, and environmental sustainability of MOF-derived materials [143].

### Metal Alloys and High-Entropy Alloys

**Fig. 13:**
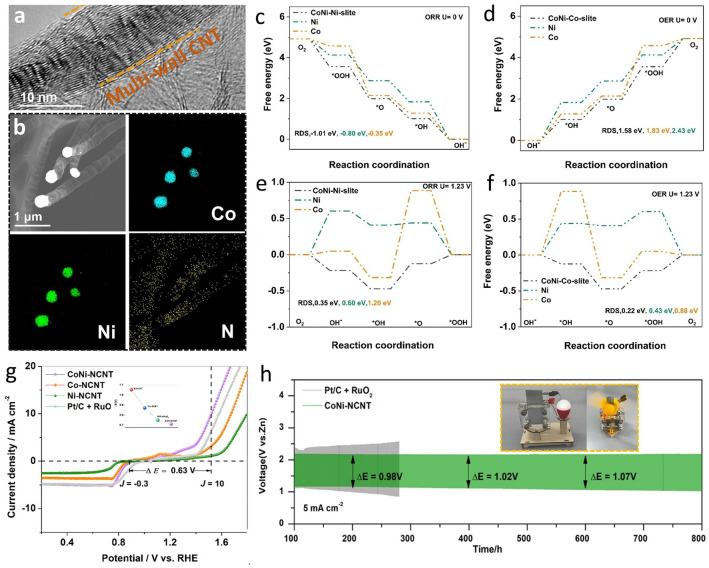
**a** HRTEM image of CNT; **b** HAADF-STEM image of CoNi-NCNT and corresponding elemental mappings of Co, Ni, and N. **c, e** Free energy diagrams for ORR at electrode potential U = 0 V and U =  − 1.23 V, respectively; **d, f** corresponding free energy diagrams for OER. **g** Polarization curves for ORR/OER. **h** Galvanostatic discharge–charge cycling curves at 5 mA cm^−2^. Reproduced with permission [54]. Copyright 2025, Elsevier

Metal alloy materials, such as those based on earth-abundant metal alloys supported on carbon nanomaterials, show promising bifunctional oxygen electrocatalytic activity [144]. Du et al. engineered a hybrid of bimetallic cobalt–nickel alloy and N-doped carbon nanotubes (CoNi-NCNT) [54]. The synthesis involved a three-step process: the catalyst precursor solution was first prepared by self-assembly, followed by in situ growth, and finally calcination, which enabled the formation of the hybrid structure (Fig. 13a). The catalyst showed homogeneous distribution of bimetallic cobalt–nickel in which the interfacial Structural microenvironment was modified through interaction with carbon nitride, promoting a synergistic effect and enhanced conductivity (Fig. 13b). The detailed theoretical calculation showed that CoNi-NCNT interfaces exhibited enhancement in electronic conductivity due to the modulation of 3*d* orbitals of the CoNi alloy by the CoN_3_ sites. As a consequence of these electronic structures’ modulation and large surface area (Fig. 13c-f), the CoNi-NCNT showed bifunctional activity for OER and ORR with a small voltage gap (Δ*E)* of 0.63 V (Fig. 13g). The ZAB fabricated using this catalyst on the air electrode showed an excellent specific capacity of 780 mAh g^−1^. This ZAB showed a long-term cycling performance of 800 h, while the ZAB fabricated using RuO_2_ and Pt/C only lasted less than 300 h (Fig. 13h).

Recently, high-entropy alloys (HEAs) have attracted much attention for their tunable composition and exceptional ability to modulate geometric and electronic structures, enabling the development of catalysts with outstanding and often unpredictable performance, making them suitable for various energy storage and conversion systems, including MABs [145]. As anticipated, the materials with multi-metal constituents exposing multiple active sites, such as HEA, enhance electrochemical redox reactions, such as the ORR and OER, and thus can be effectively used as bifunctional electrocatalysts in MAB, including Li-O_2_, Li-CO_2_, and Zn-O_2_ batteries [64]. For example, when a nanocrystalline HEA Cu–Co–Mn–Ni–Fe (CCMNF) was used as an oxygen electrocatalyst in ZAB, it showed stable performance for approximately 90 h of charging–discharging cycles [146]. This advanced performance was ascribed to the modulated electronic interaction of metal active sites constituents by the synergistic impact.

Furthermore, synthesizing complex multicomponent high-entropy nanocomposites of high-entropy alloy@oxide (HEA@HEO) was reported to tailor the bifunctional oxygen electrocatalysis. Qiu et al. developed a strategy to synthesize a HEA@HEO electrocatalyst with fourteen elements in which the PtPdAuAgCuIrRu HEA nanoclusters were anchored on AlNiCoFeCrMoTi)_3_O_4_ HEO spinel nanoporous to yield highly efficient bifunctional catalysts for ZABs [147]. This high-entropy nanocomposite exhibited a small Δ*E* of 0.61 V due to the synergism between the metal and support, making it a highly oxygen-bifunctional catalyst. One advantage of high-entropy spinel materials is that replacing one metal element does not necessarily change the structure and performance significantly. This can be advantageous, for example, when replacing rare or critical metal elements with earth-abundant ones. Toparli et al. showed that the replacement of earth-abundant divalent cations in high-entropy spinel ferrites (HESFs) did not change the crystal structure and electronic characteristics of traditional transition metal-based HESFs, such as (CoCrFeMnNi)Fe_2_O_4_ (Tm-Fe_2_O_4_) [148]. Consequently, the bifunctional (MgCoCuNiZn)Fe_2_O catalysts, in which the Mg, Cu, and Zn replaced Cr, Mn, and Fe in Tm-Fe_2_O_4_, exhibited comparable bifunctional OER/ORR performance. It also exhibited stable performance for ZAB over 200 h, depicting the importance of using earth-abundant elements yet comparable catalytic performance. Nonetheless, due to the complexity and large number of metals involved in the HEAs, it remains important to explore the active sites and the effects of these various constituents, especially for bifunctional catalytic activity.

Although there has been recent progress on HEA materials for MABs, most reported materials for LAB contain precious metal elements. These precious metal content HEAs, such as tPdIrRuAuAg and RuIrFe-CoNi HEAs, showed outstanding performance for LABs [149, 150]. In the latter, the outstanding catalytic activity was attributed to the noble metal content, while the remaining earth-abundant metals ensured high stability of this HEA [149]. Though the synthesis of HEAs is an approach to reducing the noble metal content, HEA materials free of noble metal should be explored further. Additionally, the combination of DFT and machine learning methods can be used to screen various HEA catalysts, leading to the design of highly efficient and stable OER and ORR bifunctional electrocatalysts. Sun et al., used the DFT-ML to screen the adsorption free energies of adsorbates of 729 dual-metal-site catalysts (DMSCs), and this led to the design of 30 and 11 with superior ORR and OER compared to Pt(111) and Ru_2_(110) [151]. Additionally, highly performing four bifunctional electrocatalysts (RuCoN_6_, RuIrN_6_, OsRhN_6_, and OsCoN_6_) for ORR and OER were identified.

Understanding the synergistic effect in enhancing catalytic activity is more complex in composite materials involving multiple metals, such as in high-entropy oxides (HEOs), making it difficult to determine which metals selectively influence the OER or ORR. However, the combination of DFT calculations and in situ physicochemical characterization has recently been employed to elucidate the synergistic effect and the oxygen electrocatalysis mechanism in sulfur-modified La_0.8_Sr_0.2_(CrMnFeCoNi)O_3_ high-entropy perovskite oxides (HEPOs) [152]. The introduction of sulfur modulated the microenvironmental chemistry and structure, including defects and the metal spin state, synergistically enhancing bifunctional performance. Although the lattice oxygen-mediated mechanism (LOM) predominantly governs the reaction due to the presence of oxygen vacancies, the optimized LS5M-3S sample exhibited dual reaction pathways involving both the adsorbate evolution mechanism and LOM. Through comprehensive analysis, it was concluded that Mn, Fe, Co, and Ni act as co-active sites for the OER, while Mn, Fe, and Co primarily drive ORR activity. These finding highlights that, rather than attributing improved bifunctional activity solely to a general synergistic effect, identifying specific active sites that are selectively synergistic for either OER or ORR could contribute to the design of robust bifunctional electrocatalysts.

## Conclusions and Future Directions

In the effort to develop practical MABs, the pursuit of bifunctional ECs for both ORR and OER would enable more efficient MAB designs and reduce the reliance on raw materials. This review highlights the principles of MABs and the recent developments in bifunctional ECs based on earth-abundant materials. As discussed, by optimizing both oxygen reactions, earth-abundant bifunctional ECs can allow MABs to operate with higher efficiency, longer lifespans, and better overall performance, even surpassing precious-metal-based ECs. While these recent advances in bifunctional ECs for MABs are promising, these catalysts must overcome challenges related to large-scale production, environmental sustainability of raw materials and reagents, and economic viability for widespread industrial use. The key to utilizing these ECs in future MABs lies in their long-term cyclability, compatibility with existing cathode designs, and seamless integration into battery manufacturing processes.

1. More studies are needed to better understand the microenvironment and underlying mechanisms that contribute to the bifunctional performance of OER and ORR. Since both oxygen reactions involve different mechanisms, it is critical to elucidate the origin of activity-stability in bifunctional electrocatalysts, as these materials often undergo microenvironment transformations during charging and discharging. The understanding of the active sites, degradation, synergistic or even inhibiting effect in bifunctional catalysts when active sites selectively perform specific reactions, which occurs in composites, requires more investigations.
2. The landscape of rechargeable MABs has undergone significant evolution to address the needs of various energy applications. It is also worth investigating other designs of MABs, as this could help to discover a wide range of rechargeable batteries for future sustainable energy storage. For example, a novel design for an aqueous ZAB has been proposed, leveraging the *in situ* formation and oxidation of hydrogen peroxide (HO_2_^−^) on the air electrode, termed a Zn–peroxide battery (ZPB) [153]. During discharge, the process follows a 2e^−^ pathway involving oxygen reduction and HO_2_^−^ generation on the air electrode, while the charge cycle converts HO_2_^−^ back to water and oxygen. This design demonstrates a minimal potential difference (60 mV) between ORR and peroxide oxidation due to the fast kinetics of both reactions. This design enables a reduced difference in the potential of bifunctional oxygen electrocatalysis compared to standard designs that have a large difference, making it a challenge to develop efficient bifunctional oxygen catalysts.
3. There have been significant developments in the *in situ* and *operando* characterization of ECs. However, more efforts are needed *in situ* and *operando* characterization in real-world metal–air batteries to better understand the microenvironment, activity trends, determine active sites, and assess the durability of bifunctional ECs during MAB testing. Additionally, the research in scalability, reproductivity, and stability of emerging materials, including MOF-derived materials and SACs that most involve carbon, should be prioritized to investigate the trade-offs in scalability, activity, and stability.
4. In contrast to the well-established industrial production of PGM-based catalysts, such as commercial Pt/C and Ir/C, which are already used in many different applications, the production of earth-abundant catalysts at an industrial scale is essential for their broader adoption. Currently, most synthesis of materials and physicochemical and electrochemical tests are conducted on a laboratory scale and follow protocols that are limited to this scale. This poses a significant challenge, as some of the highly efficient catalysts synthesized at the laboratory scale involve multiple steps, such as hydrothermal techniques, annealing, acid treatment, and drying in an inert environment, which, when combined, consume substantial energy even at a small scale. The cost-effectiveness of these methods, along with their environmental impact at a larger scale, should also be carefully considered. Furthermore, to deploy these ECs at a larger scale, relevant protocols for real-world applications should be established and tested.
5. Lastly, combining the experimental characterization, theoretical calculations, machine learning (ML), and artificial intelligence (AI)-guided materials development, such as AI-driven multiscale modeling methodologies, is crucial for the development of robust and highly efficient bifunctional ECs for water electrolyzer, fuel cells, and batteries, including MABs [154]. DFT combined with emerging ML tools is projected to play a crucial role in designing novel, highly efficient, durable, and scalable electrocatalysts for MABs and other electrochemical energy technologies, as it can enable the screening of many catalysts that would take more effort experimentally.
